# Autophagy buffers Ras-induced genotoxic stress enabling malignant transformation in keratinocytes primed by human papillomavirus

**DOI:** 10.1038/s41419-021-03476-3

**Published:** 2021-02-18

**Authors:** Eduardo Cararo-Lopes, Matheus H. Dias, Marcelo S. da Silva, Julianna D. Zeidler, Alexandre T. Vessoni, Marcelo S. Reis, Enrique Boccardo, Hugo A. Armelin

**Affiliations:** 1 Center of Toxins, Immune-response and Cell Signaling, Instituto Butantan, São Paulo, SP 05503-900 Brazil; 2 Department of Biochemistry, Instituto de Química, Universidade de São Paulo, São Paulo, SP 05508-000 Brazil; 3 Department of Chemical and Biological Sciences, Instituto de Biociência, Universidade do Estado de São Paulo, Botucatu, SP 18618-689 Brazil; 4 Department of Medicine, Washington University in St. Louis, St. Louis, MO 63110 USA; 5 Department of Microbiology, Instituto de Biociências, Universidade de São Paulo, São Paulo, SP 05508-900 Brazil; 6 Kogod Aging Center, Department of Anesthesiology and Perioperative Medicine, Mayo Clinic College of Medicine, Rochester, MN 55905 USA; 7 Present Address: Rutgers Cancer Institute of New Jersey, New Brunswick, NJ 08901 USA

**Keywords:** Cancer models, Macroautophagy, Stress signalling, Mechanisms of disease

## Abstract

Malignant transformation involves an orchestrated rearrangement of cell cycle regulation mechanisms that must balance autonomic mitogenic impulses and deleterious oncogenic stress. Human papillomavirus (HPV) infection is highly prevalent in populations around the globe, whereas the incidence of cervical cancer is 0.15%. Since HPV infection primes cervical keratinocytes to undergo malignant transformation, we can assume that the balance between transforming mitogenic signals and oncogenic stress is rarely attained. We showed that highly transforming mitogenic signals triggered by HRas^G12V^ activity in E6E7–HPV–keratinocytes generate strong replication and oxidative stresses. These stresses are counteracted by autophagy induction that buffers the rapid increase of ROS that is the main cause of genotoxic stress promoted by the oncoprotein. As a result, autophagy creates a narrow window of opportunity for malignant keratinocytes to emerge. This work shows that autophagy is crucial to allow the transition of E6E7 keratinocytes from an immortalized to a malignant state caused by HRas^G12V^.

## Introduction

In developing and adult organisms, a dynamically orchestrated molecular network of stress response pathways underlies the robust homeostasis of somatic cells^1^. However, uncontrolled proliferation permanently compromises the cancer cell’s robustness, overburdening its system of stress response pathways. This fragile balance between uncontrolled proliferation and the overloaded stress response is a phenotypic trait common to cancer cells^2,3^. During the onset and development of oncogenesis, cells in the transition to malignant transformation phenotype must adapt their stress response pathways to new demands of an uncontrolled proliferation surge, which lead to growing tumors or cell death.

Cervical cancer is the fourth most common cancer in women. In 2018, this disease affected 570,000 women killing 311,000^4^. Prior HPV infection renders cervical keratinocytes prone to malignant transformation driven by Ras oncogene mutations^5^. Ras is a small GTPase responsible in connecting the signal transduction from membrane tyrosine kinase receptors to protein kinase cascades^6^. The KRas isoform is found in 75% of all cases of cancer in which Ras is mutated^7,8^; nevertheless, in HPV-primed keratinocytes, leading to head–neck and cervical cancers, HRas plays an important role^9–11^.

Although HPV is the most common sexually transmitted disease, only a small proportion of infected women develop cervical cancer or its precursor lesions^4,5^. This implies that, in vivo, human HPV-primed keratinocytes must undergo severe oncogenic stress during oncogenesis. To experimentally analyze this phenomenon, we evaluated a cell culture model of malignant transformation. As a first and necessary step, primary cultures of human keratinocytes were previously immortalized by constitutive expression of E6E7/HPV16 genes, which promote degradation of p53 and pRb proteins. Posteriorly, the E6E7 keratinocytes were subjected to malignant transformation triggered by HRas^G12V^ expression^12–14^. However, the molecular mechanisms underlying the transition from immortalized to malignant transformed E6E7/HPV keratinocytes were not identified, particularly the role of autophagic pathways^15^.

Autophagy is a prosurvival mechanism responsible for the degradation and recycling of cytosolic contents, including protein complexes and organelles^16^. This process can provide substrates to keep cellular homeostasis in cases of high energetic and anabolic demand regularly found during oncogenesis^17^ (e.g., to maintain nucleotide pools^18^). Moreover, autophagy promotes clearance of dysfunctional mitochondria in cancer cells preventing the accumulation of reactive oxygen species (ROS)^19^. Nonetheless, uncontrolled autophagic flux can also promote cell death^20–23^.

Here, we report quantitative analyses of a cell culture model consisting of an E6E7/HPV-immortalized human keratinocyte (hereafter E6E7 keratinocytes) conditionally subjected to ER:HRas^G12V^ expression upon 4OH-tamoxifen (4OHT) induction. This experimental design allowed fine-tuning modulation of estrogen-receptor-inducible form ER:HRas^G12V^ activity, during the transition from immortalized to malignant transformed E6E7 keratinocytes.

## Results

### High HRas^G12V^ activity caused severe oncogenic stress in E6E7 keratinocytes

We transduced E6E7 keratinocytes with a constitutively expressed HRas^G12V^ oncogene construction aiming to probe into a potential transformation barrier in this type of human cells. Four days after HRas^G12V^ transduction, cells showed remarkably high levels of HRas^G12V^ and P-ERK1/2 compared with control cells transduced with empty vector control (EV) (Supplementary Fig. S1A). In addition, HRas^G12V^-transduced cultures displayed aberrant morphology with several vacuoles, low proliferation, and loss of viability (Supplementary Fig. S1B). However, 30 days after transduction, HRas^G12V^ and P-ERK1/2 levels markedly decreased in HRas^G12V^-transduced cells, and signs of crisis were no longer observed. Moreover, by this time, HRas^G12V^-transduced cells showed higher proliferation rates and increased saturation density compared with EV (Supplementary Fig. S1C, S1D). These results suggested that high HRas^G12V^ levels in E6E7 keratinocytes cause strong oncogenic stress, and may lead to the selection of low-expressing HRas^G12V^ keratinocytes with malignant traits.

**Fig. 1:**
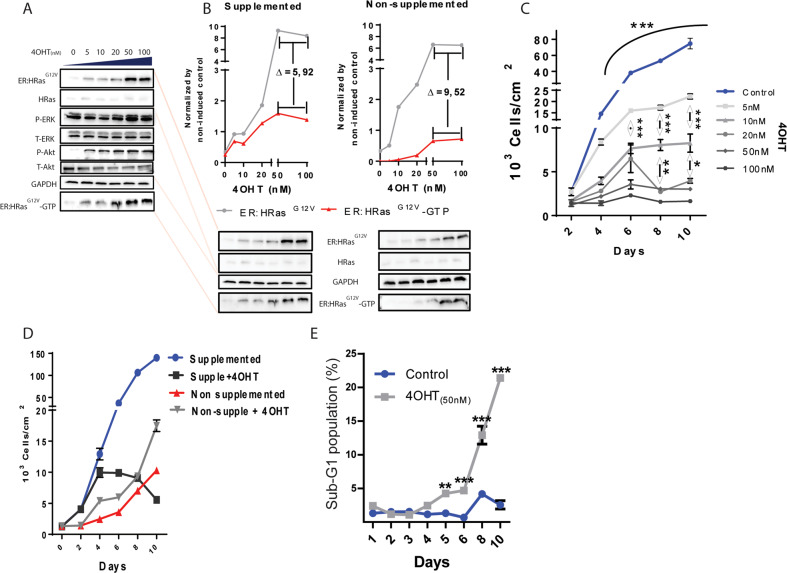
High HRas^G12V^ activity causes oncogenic stress in the E6E7 keratinocytes grown in supplemented media. **A** Immunoblots: 4OHT dose–response of ER:HRas^G12V^ induction. The activity of HRas^G12V^ was monitored by a RAS-GTP-binding assay, which leads to downstream activation of P-ERK and P-Akt. **B** Immunoblots comparing ER:HRas^G12V^ levels between supplemented (5 ng/mL of EGF and 5 µL/mL of pituitary extract) and nonsupplemented media in ER:HRas^G12V^-keratinocyte cultures. The immunoblot of cells in the supplemented medium is a projection of the one presented in **A**. The electrophoresis gel of cell lysates derived from cells grown in either supplemented or nonsupplemented media, was blotted in the same membrane to allow comparison of ER:HRas^G12V^ levels and HRas^G12V^-GTP (HRas^G12V^-GTP = HRas^G12V^ activity) under both conditions. **C** Growth curves of ER:HRas^G12V^ keratinocytes in function of 4OHT concentration. The graph is representative of three independent experiments carried out in duplicate. **D** Effect of 50 nM 4OHT in ER:HRas^G12V^ keratocytes growing in supplemented or nonsupplemented media. The graph is representative of three independent experiments carried out in duplicate. **E** Evolution of the sub-G1 population of ER:HRas^G12V^ keratocytes growing in complete medium induced or not with 50 nM of 40HT, measured by flow cytometry after PI staining. The graph is representative of two independent experiments. For flow cytometry, 30 × 10^3^ events were considered per condition and time point. Growth curves and the sub-G1 population are presenting data as mean (SD). Two-way ANOVA *P ≤ 0.05, **P ≤ 0.01, and ***P ≤ 0.001, Bonferroni post hoc.

We transduced the ER:HRas^G12V^ in E6E7 keratinocytes, yielding the inducible sublineages (SLis) (Fig. 1A and Supplementary Fig. S1E, S1F). 4OHT dose–response curves showed ER:HRas^G12V^ expression increasing from negligible levels to a plateau at 50 nM of 4OHT (Fig. 1A). The activation of the downstream proteins ERK1/2 and Akt also peaked at 50 nM. To check whether the HRas^G12V^ activity was dependent on an external growth factor, we induced ER:HRas^G12V^ keratinocytes in two different media compositions, with and without EGF and pituitary extract supplementation. HRas^G12V^ activity in the form of ER:HRas^G12V^–GTP concentration was shown to be dependent on growth factor supplementation (Fig. 1B).

In complete media, doses of the inductor ranging from 5 to 100 nM impaired cell proliferation in a dose-dependent manner (Fig. 1C). Moreover, the cytomorphological signs of crisis were also dose-dependent on HRas^G12V^ activity (Supplementary Fig. S2A). Interestingly, in nonsupplemented culture media, the addition of 50 nM of 4OHT caused a slight growth increase over 10 days (Fig. 1D), whereas this concentration of the inductor blocked cell proliferation and induced cell death by day four in complete media (Fig. 1D, E). These results showed that E6E7 keratinocytes are toxically sensitive to high levels of HRas^G12V^ activity.

ER:HRas^G12V^ keratinocytes maintained in 20, 50, and 100 nM of 4OHT were all dead in 3–6 weeks (Supplementary Fig. S3A). However, cells maintained in 5 and 10 nM of 4OHT were resistant to HRas^G12V^-inducible activity for 6 months. These survivor sublineages (LTE5 and LTE10) exhibited a relative ER:HRas^G12V^-induction ceiling about tenfold lower than their parental inducible ER:HRas^G12V^ keratinocytes (Supplementary Fig. S3B). Moreover, it is noteworthy that after this long-term selection, ER:HRas^G12V^ induction with 5–50 nM of 4OHT no longer caused cytotoxicity in the LTE5 (Supplementary Fig. S3C), it rather increased saturation density (Supplementary Fig. S3D), contrary to the inducible parental lineage ER:HRas^G12V^ keratinocytes (Supplementary Fig. S3E). Altogether, these results suggested that high HRas^G12V^ activity was a barrier hardly tolerated during the malignant transformation transition of E6E7 keratinocytes.

### In E6E7 keratinocytes, high levels of HRas^G12V^ activity cause cell cycle arrest and late cell death

**Fig. 2:**
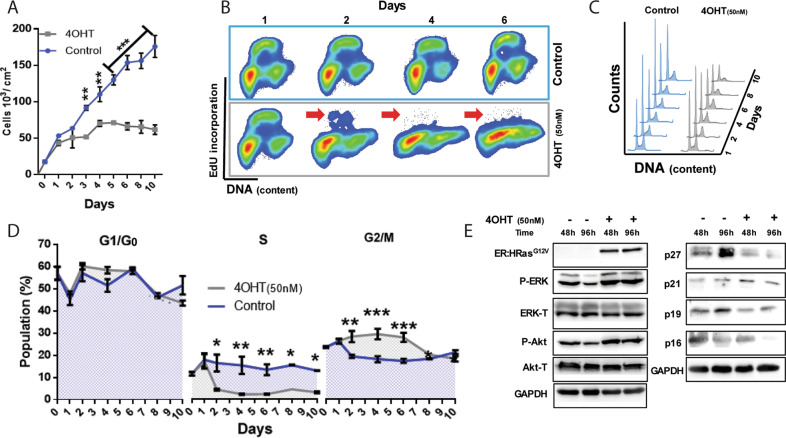
Oncogenic stress induced by high HRas^G12V^ activity in E6E7 keratocytes causes cell cycle arrest at G1/G0 and G2/M phases. **A** Growth curve of ER:HRas^G12V^ keratinocytes in the supplemented medium induced or not with 50 nM of 4OHT. The graph is representative of three independent experiments carried out in triplicate. **B** Cell cycle profile analyses of growing ER:HRas^G12V^ keratinocytes, using 1-h pulse of EdU incorporation and propidium iodide (PI) staining, showed a progressive decrease in DNA synthesis (red arrows) in cells induced with 50 nM 4OHT. **C** DNA content profiles of growing ER:HRas^G12V^ keratinocytes stained with PI, with or without 4OHT induction. **D** Steady-state growing ER:HRas^G12V^ keratinocytes were induced or not with 50 nM 4OHT at zero time: kinetics of the relative size of cell cycle-phase subpopulations; evidence of cell cycle arrest at G1/G0 and G2/M phases only in 4OHT-induced cells. The graph is representative of four independent experiments, two PI single-labeled, and two PI plus EdU double-labeled. All conditions and time points were carried out in duplicate. **E** Immunoblots showed intracellular levels of critical components of cell cycle control pathways in growing ER:HRas^G12V^ keratinocytes induced or not with 4OHT. For flow cytometry, 30 × 10^3^ cells were considered per condition and time point. Data presented as a mean (SD). Two-way ANOVA *P ≤ 0.05, **P ≤ 0.01, and ***P ≤ 0.001, Bonferroni post hoc.

The cell proliferation inhibition caused by HRas^G12V^ activity in this ER:HRas^G12V^ keratinocytes model (Fig. 2A) likely involved cell cycle arrest. We pulse-labeled DNA and analyzed the cell cycle dynamics (Fig. 2B–D). The results showed that G1 → S transition was blocked after 24 h of 4OHT induction since the S-phase subpopulation plummeted to negligible size by day 2. However, the increased number of 4OHT-induced cells arrested in G2/M indicated an additional cell cycle blockage. Intriguingly, immunoblotting results showed no alterations in the levels of physiological inhibitors of G1-phase progression (p19 and p16) in these 4OHT-induced cells (Fig. 2E).

**Fig. 3:**
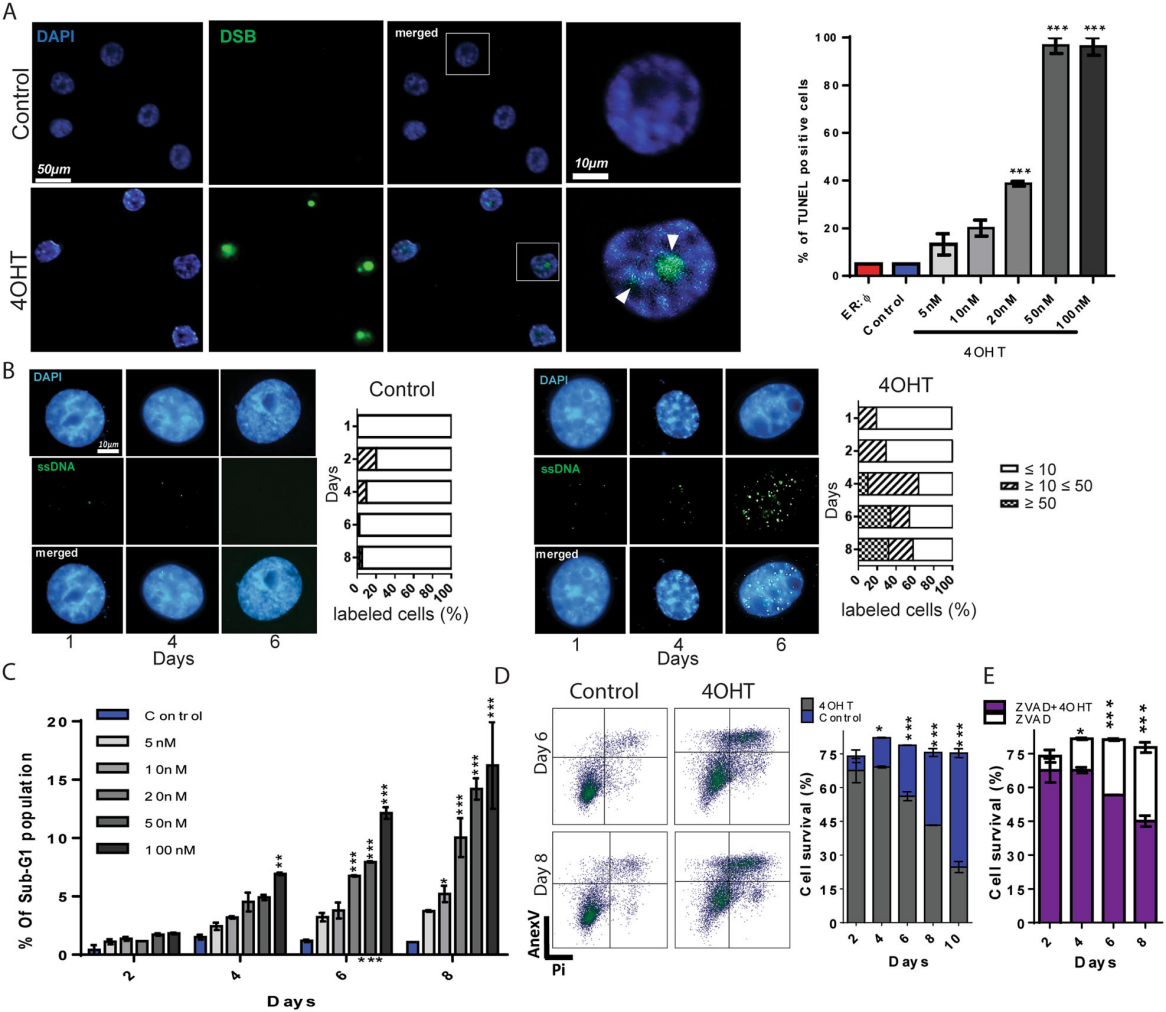
The oncogenic stress triggered by high HRas^G12V^ activity causes DNA damage leading to cell death. **A** TUNEL assays showed high levels of DNA strand breaks (DSB) in cells after six days of 4OHT induction in a dose-dependent manner. Two groups of controls were used: noninduced ER:HRas^G12V^ keratinocytes and ER:ø-keratinocytes treated with 50 nM of 4OHT emphasizing that the increasing levels of HRas^G12V^, but not 4OHT, are responsible for DNA breaks, *n* = 100 cells per condition. The whole experiment (including the experimental controls and the other 4OHT concentrations used), is presented in Supplementary Fig. S4. **B** In ER:HRas^G12V^ keratinocytes, native chromatin-BrdU assay showed persistent sites of single-stranded DNA (ssDNA) after six days of 50 nM 4OHT induction. For each condition and day analyzed, at least 50 nuclei were counted and classified into three different phenotypes: normal ≤10 foci/nucleus, the initial stage of replication stress ≥10 ≤ 50 foci/nucleus, and advanced stage of replication stress ≥50 foci/nucleus. The whole kinetic experiment is displayed in Supplementary Fig. S5. **C** Sub-G1 populations increased with dose and time of HRas^G12V^ induction. The graph is representative of two independent experiments carried out in duplicate. **D** Annexin-V/PI-flow cytometry analyses: ER:HRas^G12V^ keratinocytes induced with 50 nM of 4OHT and the noninduced control condition were analyzed for 10 days. Density plots are presented as an example of the profile of ER:HRas^G12V^ keratinocytes in days 6 and 8. **E** In the same kinetic design, the treatment with 25 µM of the caspase inhibitor ZVAD did not rescue the viability of keratinocytes under high HRas^G12V^ activity. The graphs are representative of three independent experiments. For flow cytometry, 30 × 10^3^ cells were considered per condition and time point. Data presented as a mean (SD). One-way ANOVA for **B** and two-way ANOVA for **C** and **D.** *P ≤ 0.05, **P ≤ 0.01, and ***P ≤ 0.001, Bonferroni post hoc.

The G2/M arrest suggested the presence of DNA damage caused by the HRas^G12V^-induced activity. To evaluate the presence of DNA damage in ER:HRas^G12V^ keratinocytes, we carried out a TUNEL assay. We found that on the 6th day of induction with 50 nM of 4OHT, cells displayed nearly 100% of TUNEL-positive cells (Fig. 3A and Supplementary Fig. S4).

A possible explanation for DNA breaks observed in ER:HRas^G12V^ keratinocytes is due to an over mitogenic signal triggered by high HRas^G12V^ activity. High proliferative rates enhance the replication stress present in tumor cells^3,24^. This could lead to genomic instability and DNA breaks if the replication stress response is not able to completely repair the DNA damage, such as exposure of long tracts of single-stranded DNA (ssDNA), which is a primary signal of this type of stress^25^. Thus, to verify the presence of replication stress, we measured the ssDNA exposure through a native-BrdU immunofluorescence assay^26^. We found an increased number of BrdU foci in DNA in 4OHT-induced cells from days 1 to 8 (Supplementary Fig. S5); by day 6, 30% of cells displayed more than 50 BrdU foci per cell; on the contrary, uninduced cells exhibited negligible basal levels of BrdU foci (Fig. 3B).

Kinetic flow cytometry analyses of keratinocytes under high HRas^G12V^ activity showed cell death increase in a dose-dependent manner from day 4 to day 10 of induction (Fig. 3C). Furthermore, annexin-V/PI double-stained profiles of cells induced with 50 nM of 4OTH for up to 10 days confirmed cell death (Fig. 3D), which was not rescued by the treatment with the caspase inhibitor ZVAD (Fig. 3E), showing that the cell death is independent of caspase activity. Thus, these results presented the progressive oncogenic stress crisis triggered by HRas^G12V^ overactivity in E6E7 keratinocytes, which comprises replication stress, cell cycle arrest, and late cell death.

### Oxidative stress underpinned the damaging effects of high HRas^G12V^ activity in E6E7 keratinocytes

DCF fluorescence labeling indicated a HRas^G12V^ dose-dependent elevation of ROS in induced ER:HRas^G12V^ keratinocytes (Supplementary Fig. S6A). In addition, MitoSOX fluorescence labeling evidenced mitochondrial superoxide accumulation in induced cells (Supplementary -Fig. S6B). These results confirmed that HRas^G12V^ activity induced oxidative stress in E6E7 keratinocytes, suggesting a causal role in the oncogenic stress crisis described.

**Fig. 4:**
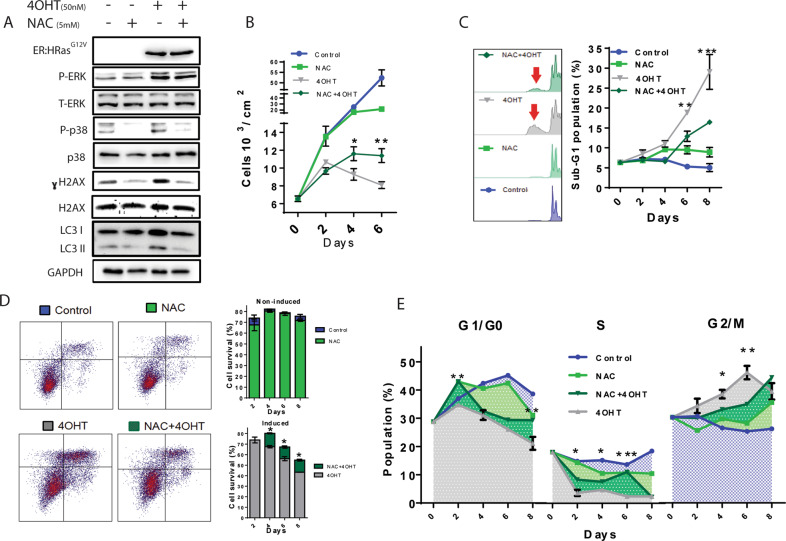
The antioxidant NAC mitigated the toxic effects of HRas^G12V^ activity, diminishing DNA damage and increasing cell viability. **A** Immunoblots of ER:HRas^G12V^ keratinocytes induced for 48 h with 50 nM 4OHT, treated or not with 5 mM of NAC. **B** Growth curves of ER:HRas^G12V^ keratinocyte induced or not, showing the protective effect of NAC on cell proliferation. The graph is representative of two independent experiments carried out in duplicate. **C** DNA content by PI-flow cytometry analyses showed that NAC increased cell viability, evidenced by the elimination of the sub-G1 population on the 8th day of induction. The graph is representative of two independent experiments. **D** Annexin-V/PI-flow cytometry cell viability assays: quantified density plot graphs also demonstrated NAC survival effect on ER:HRas^G12V^ keratinocytes induced with 4OHT. Density plots are presenting the profile of keratinocytes treated or not with 4OHT on their 8th day. The graph is representative of two independent experiments. **E** PI-flow cytometry cell cycle analyses showed that NAC-treated ER:HRas^G12V^ keratinocytes could overcome the G1 and G2/M cell cycle arrest caused by high HRas^G12V^ activity. The significance presented in the graph is related to the comparison between cells induced with 50 nM of 4OHT with (dark green) or without (gray) NAC treatment. The graph is representative of two independent experiments. For flow cytometry, 30 × 10^3^ cells were considered per condition and time point. Data presented as mean (SD). Two-way ANOVA *P ≤ 0.05, **P ≤ 0.01, and ***P ≤ 0.001, Bonferroni post hoc.

Treatment with the ROS scavenger NAC (N-Acetyl cysteine) reduced the levels of single-stranded DNA accumulation induced by 50 nM 4OHT in ER:HRas^G12V^ keratinocytes (Supplementary Fig. S7). The phosphorylation of both p38 and H2AX was also decreased in the presence of NAC, with no changes in the ERK activation induced by HRas^G12V^ activity (Fig. 4A). These results confirmed that HRas^G12V^ activity induced oxidative stress in E6E7 keratinocytes, which account for the oncogenic stress crisis described and indicate that NAC attenuates the replication stress/DNA damage induced by high HRas^G12V^ activity without affecting its mitogenic signaling. NAC alleviated the cell cycle arrest induced by HRas^G12V^ activity, partly relieving the cell proliferation inhibition (Fig. 4B). Moreover, NAC partially rescued ER:HRas^G12V^ keratinocytes from the cell death triggered by high HRas^G12V^ activity, as shown by sub-G1 subpopulation measurements (Fig. 4C) and annexin-V/PI assays (Fig. 4D). Altogether, these results show that oxidative stress was a major cause of the oncogenic stress crisis triggered by high HRas^G12V^ activity in E6E7 keratinocytes, which act in the cell cycle blockages at G1 → S transition and G2/M phase (Fig. 4E).

### Autophagy buffers oncogenic stress triggered by HRas^G12V^ activity creating a survival and proliferative window for malignant keratinocyte surges

**Fig. 5:**
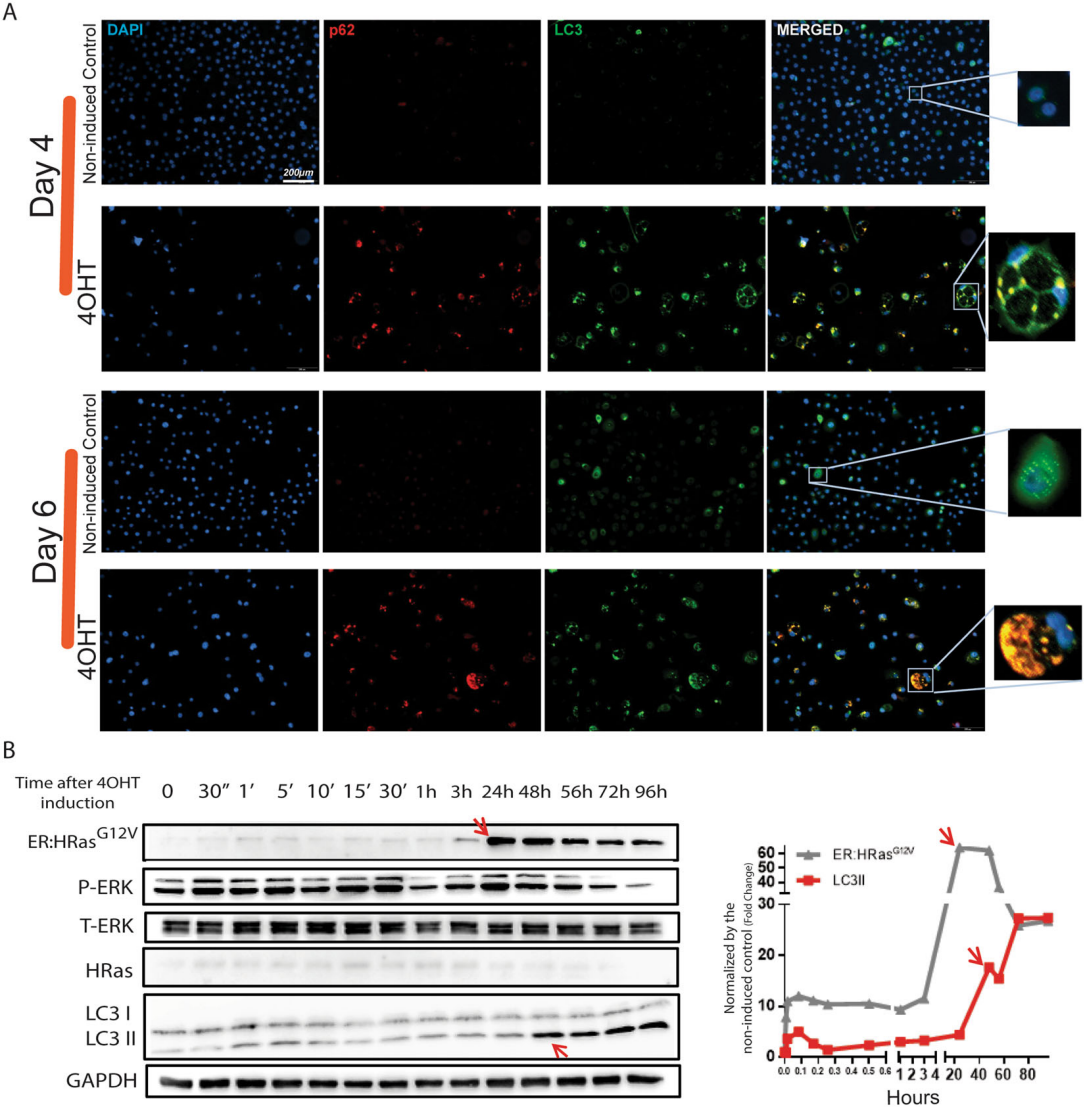
Autophagic flux was increased 48 h after HRas^G12V^ induction. **A** Forty-eight hours after HRas^G12V^ induction, immunofluorescence assays (IFA) exhibited the onset of the two autophagic markers LC3 and p62, which colocalized the surrounding vacuoles, suggesting a cause-and-effect relationship between HRas^G12V^ activity and autophagy initiation. **B** The kinetics of this phenomenon was further clarified by immunoblots that showed HRas^G12V^ induction at 24 h followed by LC3II expression at 48 h (see red arrows), implying that the autophagy initiation was in response to the oncogenic stress caused by HRas^G12V^ activity.

Induced ER:HRas^G12V^ keratinocytes microscopically displayed vacuoles that progressively increased in size and quantity depending on 4OHT concentration and time of induction (Supplementary Fig. S2). Immunofluorescence evidenced the colocalization of autophagic proteins p62 and LC3 at days 4 and 6 (Fig. 5A) after HRas^G12V^ induction. In addition, immunoblotting kinetics showed increased levels of LC3II, an indicator of autophagic activity, only after the peak of 4OHT-induced HRas^G12V^ expression (Fig. 5B).

**Fig. 6:**
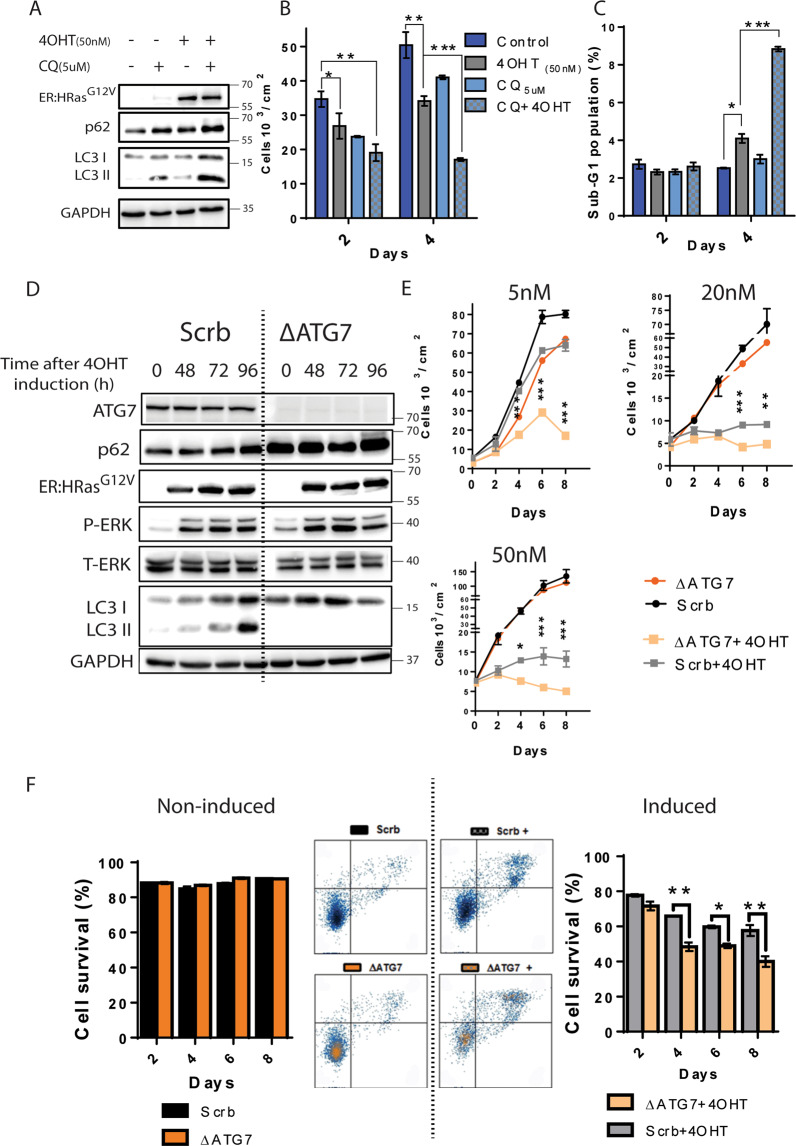
Autophagy promoted survival in keratinocytes under oncogenic stress triggered by HRas^G12V^ activity. **A** Immunoblots of ER:HRas^G12V^ keratinocytes presenting autophagy markers (LC3II and p62) under 48 h of CQ (5 µM) treatment induced or not with 4OHT (50 nM). **B** The growth curve of induced ER:HRas^G12V^ keratinocytes suggests loss of cell viability of keratinocytes that had their autophagy flux inhibited. The graph is representative of three independent experiments carried out in duplicate per condition and time point. **C** DNA content analysis by flow cytometry using PI of labeled ER:HRas^G12V^ keratinocytes showed a drastic increase of sub-G1 population in 4OHT-induced keratinocytes treated with CQ. The graph is representative of two independent experiments. **D** Immunoblots of ER:HRas^G12V^ autophagy-defective keratinocytes (ΔATG7 keratinocytes) and their control autophagy-competent scrabble (Scrb keratinocytes). The absence of ATG7 and LC3II in ΔATG7 keratinocytes induced with 50 nM 4OHT confirmed successful knockout of the ATG7 gene and ablation of the autophagy pathway. **E** Growth curves using different 4OHT concentrations and consequently different levels of HRas^G12V^ activity. The graph is representative of three independent experiments carried out in duplicate. **F** Flow cytometry of cell viability assay stained with annexin-V/PI. Density plot graphs (8th day) and their quantification indicate that autophagy activity is crucial for keratinocyte survival under oncogenic stress promoted by HRas^G12V^ activity. The graph is representative of two independent experiments in which 30 × 10^3^ cells were considered per condition and time point. Data presented as a mean (SD). Two-way ANOVA, *P ≤ 0.05, **P ≤ 0.01, and ***P ≤ 0.001, Bonferroni post hoc.

These results showing autophagy upregulation following the increase in the oncogenic HRas^G12V^ levels suggested that autophagy was triggered in response to HRas^G12V^ activity potentially to keep the cell viability. To further investigate the role of autophagy in the induced ER:HRas^G12V^ keratinocytes, we used hydroxychloroquine (CQ), an autophagy inhibitor. Immunoblotting for LC3II revealed that 48 h of HRas^G12V^ induction increased autophagic initiation but when combined with CQ treatment also increased the accumulation of nonprocessed autophagic labeled substrates, as shown by the accumulation of p62, suggesting that HRas^G12V^ activity surpassed the autophagic flux limit (Fig. 6A)^27,28^. After 96 h of HRas^G12V^ induction combined with CQ, both the proliferation inhibition (Fig. 6B) and cell death (Fig. 6C) drastically enhanced.

We established an ER:HRas^G12V^ keratinocyte sublineage defective in autophagy by knocking out the gene encoding the protein ATG7 using the CRISPR–Cas9 system. In contrast with the scramble control cells (hereafter Scrb keratinocytes), the resultant knocked-out sublineage (hereafter ∆ATG7 keratinocytes) showed undetectable levels of ATG7 and no autophagy activation when induced (Fig. 6D). Upon HRas^G12V^ induction, ∆ATG7 keratinocytes failed to process LC3I–LC3II, and the p62 levels were increased compared to Scrb keratinocytes, confirming successful ablation of the autophagic pathway. In addition, HRas^G12V^ induction and MAPK–ERK1/2 activation were similar in Scrb- and ∆ATG7 keratinocytes, showing that the pattern of HRas^G12V^ induction and mitogenic activation were preserved. ∆ATG7 keratinocytes turned out to be extremely sensitive to the oncogenic stress initiated by HRas^G12V^ activity. Growth curves showed that the proliferation of ∆ATG7 keratinocytes was strongly inhibited even by 5 nM of 4OHT induction, which was well tolerated by parental ER:HRas^G12V^ keratinocytes or Scrb keratinocytes (Fig. 6E, 5 nM). This increased sensitivity to HRas^G12V^ activity in ∆ATG7 keratinocytes was even more pronounced in higher concentrations of 4OHT (Fig. 6E, 20 and 50 nM). Moreover, annexin-V/PI assays demonstrated that ∆ATG7 keratinocytes under induction died earlier and at higher rates than Scrb keratinocytes (Fig. 6F).

**Fig. 7:**
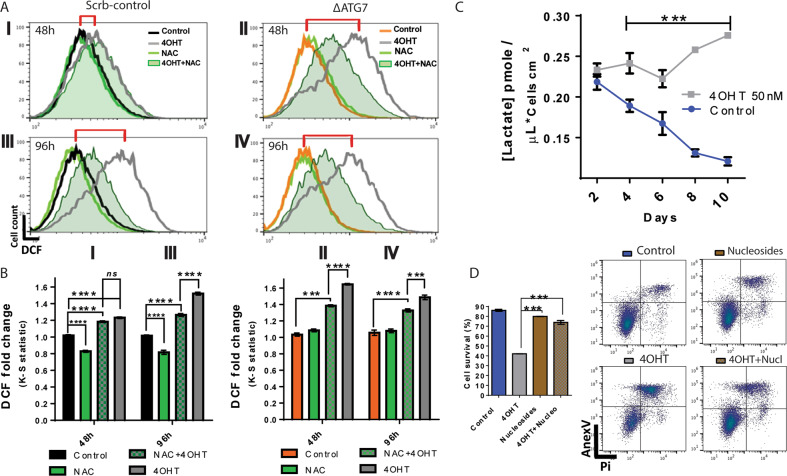
Autophagy buffers the levels of ROS, reducing the DNA damage caused by oxidative species, hence increasing the survival of keratinocytes transitioning to a malignant transformed phenotype. **A** Flow cytometry measurements of reactive oxygen species (ROS) through DCF fluorescence. Autophagy-competent (Scrb keratinocytes I–III) and autophagy-deficient keratinocytes (ΔATG7 keratinocytes II–IV) were induced with 50 nM 4OHT and treated with 5 mM NAC for 48 h (I, II) or 96 h (III, IV). The red brackets show the gradual increase in ROS levels in autophagy-competent cells and the fast increase of ROS levels in keratinocytes lacking autophagy. **B** ΔATG7 keratinocytes increase in 70% of their ROS levels after 48 h of HRas^G12V^ induction. The levels in autophagy-competent keratinocytes were much lower (20%). In addition, NAC treatment reduced steady-state levels of ROS in noninduced keratinocytes. The dislocation of the fluorescent population was quantified by the Kolmogorov–Smirnov test (K–S). The graphs are representative of three independent experiments. **C** The conditioned culture media of ER:HRas^G12V^ keratinocytes, 4OHT-induced and noninduced (control), were harvested for 10 days and had their lactate concentration measured. The graph is representative of two independent experiments carried out in triplicate. **D** The flow cytometry of cell viability assay stained with annexin-V/PI showed that deoxynucleoside treatment improved keratinocytes’ survival under oncogenic stress promoted by HRas^G12V^ activity. The ER:HRas^G12V^ keratinocytes were plated in lower density to extend as much as possible the experiment. After 6 days, the cells were induced with 50 nM 4OHT, treated with 2 mM deoxynucleosides (deoxyadenine, -guanosine, -cytidine, and thymidine) and their respective controls had their viability measured. The graph is representative of two independent experiments. The different font sizes represent the intensity of the effect. For flow cytometry, 30 × 10^3^ cells were considered per condition and time point. Data presented as a mean (SD). Two-way ANOVA, *P ≤ 0.05, **P ≤ 0.01, and ***P ≤ 0.001, Bonferroni post hoc.

The results of Figure 4 indicated that ROS generation was the main cause of oncogenic stress triggered by HRas^G12V^ activity. These results also suggested that autophagy upregulation was a prosurvivor response, aiming to keep cell viability. Furthermore, immunoblot showed that HRas^G12V^-induced keratinocytes displayed reduced levels of autophagy when cells were treated with NAC (Fig. 4A, LC3II). We compared the levels of ROS between ∆ATG7 keratinocytes and Scrb keratinocytes. By 48 h of induction, HRas^G12V^ activity increased ROS levels in about 20% in the Scrb keratinocytes (Fig. 7AI, BI); markedly, in ∆ATG7 keratinocytes, HRas^G12V^ activity resulted in over 70% increase (Fig. 7AII, BII). Such elevation in the ROS levels was not found in autophagy-competent keratinocytes, even in longer time points after induction (Fig. 7AIII, BIII). Further linking the oxidative stress to the observed autophagy, NAC treatment partially prevented ROS acute increase in ∆ATG7 keratinocytes, protection even higher than in Scrb keratinocytes after HRas^G12V^ induction, showing that the ROS-buffering effect was dependent on autophagic activity (Figs. 7A, BI, BII).

Besides the autophagy-buffering effects on ROS, elevated glycolytic levels suggested that autophagy could also be recruited in consequence of a metabolic imbalance in keratinocytes upon high HRas^G12V^ activity (Fig. 7C). In these conditions, autophagy can be mobilized to relieve this metabolic imbalance by supplying cells with essential substrates^18,29^. Thus, we fed induced ER:HRas^G12V^ keratinocytes with deoxynucleosides for 6 days. Deoxynucleoside feeding rescued ER:HRas^G12V^ keratinocytes from death but did not recover cell proliferation, suggesting that nucleotide pool deregulation was part of the oncogenic stress triggered by high HRas^G12V^ activity (Fig. 7D).

**Fig. 8:**
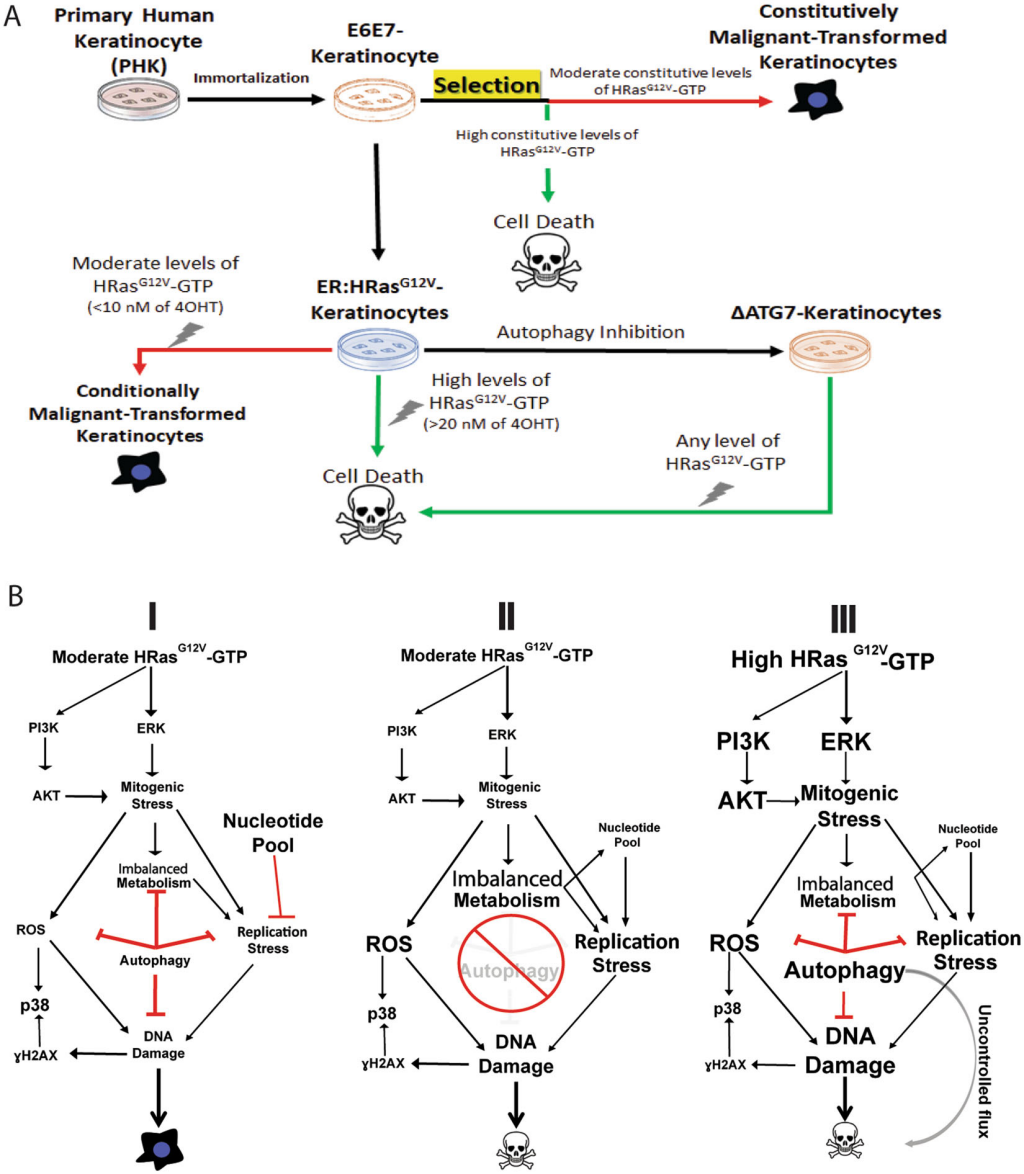
HPV-primed keratinocytes require autophagy to counteract the oncogenic stress triggered by HRas^G12V^ activity during the malignant transformation transition. **A** Step-by-step of the models generated and the role of HRas^G12V^ activity in their fates. Note that the different levels of inducible ER:HRas^G12V^ activity are mimicking the fates observed in the constitutive ones. In the constitutive model, we could observe only the product of a random selection of cells presenting low levels of HRas^G12V^ activity. Nonetheless, using the inducible model, we were able to observe the phenomenon responsible for such selection. **B** Mechanistic model presenting the challenge that HPV-primed keratinocytes, in the transition to a malignant transformed phenotype, must bypass according to the intensity of oncogenic stress (ROS, imbalanced metabolism, replication stress, and DNA damage). The intensity of the oncogenic stress is determined by different levels of HRas^G12V^ activity and it is counterbalanced by autophagic activity in a different fashion: (I) only a moderate level of HRas^G12V^ activity is supported by immortalized E6E7 keratinocytes since the autophagy activity, also moderate, is capable of buffering the oncogenic stress generated. (II) However, without the recruitment of autophagy, any level of oncogenic stress is not tolerated, proving that autophagy is recruited primarily as a safeguard mechanism. (III) On the other hand, high levels of HRas^G12V^ activity generate unbearable oncogenic stress followed by the activation of autophagy over the cell capacity of processing that becomes an additional source of stress leading to cell death. The different font sizes are expressing the levels of cell signaling/events observed.

Altogether, these results revealed that autophagy prevents HRas^G12V^-dependent rapid ROS generation, opening a window for survival and proliferation of E6E7 keratinocytes under stress caused by high HRas^G12V^ activity. This window would allow the selection of malignant HPV-primed keratinocytes, making autophagy a tumor promoter agent in this context (Fig. 8).

## Discussion

Mutated HRas is found in approximately 230,000 cases of cancer globally^11^. However, as we have shown, the oncogenic potential of mutated HRas is limited by its own activity. We demonstrated that different levels of HRas^G12V^ activity are responsible for different cell fates, which were determined by the oncogenic stress levels tolerated by keratinocytes. In E6E7 keratinocytes, high HRas^G12V^ activity initiated a chain of stresses, leading to discoordination of the mitogenic signaling that propagates to replication impairment and oxidative stress, both responsible for DNA damage.

We demonstrated that elevated HRas^G12V^ activity strongly signaled cell cycle progression as indicated by MAPK stimulation and p16 and p19 inhibition (Fig. 2D, C). Paradoxically, 48 h after induction rather than progressing, the keratinocytes remained blocked in the G1/G0 phase (Fig. 2), exhibiting symptoms of mitogenic stress. Ras is a causative agent of mitogenic stress since it can deregulate cell cycle checkpoints through the Raf/MEK/ERK axis^30,31^. Besides, sustained Ras activity can mediate genomic instability^32^. In this scenario, genomic instability was caused by defects in the spindle assembly checkpoints that became an important source of DNA damage signaling to p53, which resulted in interruption of cell cycle progression and potentially in cell death^32^. In lung cells, the transition from hyperplastic to malignant phenotype is associated with genomic instability that positively selects cells with p53-inactivating mutations^33^. Furthermore, functional p53- signaling is the base to explain the better prognostics found in HPV-head–neck cancers in comparison with HPV + ones from the same type^34,35^.

In the case of E6E7 keratinocytes, p53 and pRb were degraded by E6E7/HPV proteins (Supplementary Fig. S1G, S1H, S1I); however, these cells under high HRas^G12V^ activity were driven to cell cycle arrest followed by death. We hypothesize that the genomic instability due to sustained high HRas^G12V^ activity is the cause of a genomic crisis that typically occurs in cells transitioning to the malignantly transformed stage that is characterized by mitotic arrest, hyperstimulation of autophagy, and cell death^15,36^, events systematically present in E6E7 keratinocytes under high HRas^G12V^ activity as we have shown (Figs. 2 and 5). Moreover, the degradation of p53 (Supplementary Fig. S1H) and the high index of cell death under high HRas^G12V^ activity, even when the caspase inhibitor ZVAD is used (Fig. 3E), suggested that the genomic crisis allied to high levels of ROS might be responsible for a nonapoptotic cell death independent on caspase activation. Besides, annexin-V/PI assays showed that the wide majority of nonviable keratinocytes are double-labeled (Figs. 3D, 4D, and 6F), which is consistent with abrupt rupture of the cytoplasmatic membrane that is one of the main hallmarks of necrosis^37^. To conclude, the high levels of ROS (Fig. 6), the rescue of cell viability using the ROS scavenger NAC, and the potential metabolic imbalance (Figs.4 and 7C/D) allow us to propose that ferroptosis, a specific form of regulated necrosis^38^, might have been trigged as an additional tumor suppressor mechanism that superposes the deficiency of canonical checkpoint signaling when E6E7 keratinocytes are exposed to high HRas^G12V^ activity, but further evidence is necessary to support such proposal.

Another source of DNA damage caused by high HRas^G12V^ activity is the replication stress that could be compounded by different molecular events: increased firing origin of replication, impaired fork progression^39,40^, depleted nucleotide pool^41^, and stimulated replication–transcription conflicts^42^. Nonetheless, our findings suggested that the predominant factor that led to replication impairment and DNA damage was oxidative stress. We demonstrated that E6E7 keratinocytes under high HRas^G12V^ activity treated with NAC considerably reduced the levels of ssDNA and γH2AX (Supplementary Fig. S7 and Fig. 4A), allowing these cells to overcome the G1/G0 blockage, to progress throughout the S phase, and delay the blockage in G2/M (Fig. 4E).

We showed that the limited improvement observed in NAC treatment was linked to autophagic activity as suggested by LC3II- and ROS-reduced levels in induced keratinocytes treated with NAC (Figs. 4A and 7A/B). Autophagy buffers the acute generation of ROS caused by HRas^G12V^ activity enhancing the keratinocyte viability. In autophagy-competent E6E7 keratinocytes (Scrb keratinocytes), the ROS levels increased but never reached the threshold of autophagy-deficient E6E7 keratinocytes (ΔATG7 keratinocytes). We hypothesized that the main source of the observed ROS is mitochondria, as suggested by the increased levels of mitochondrial superoxide (Supplementary Fig. S6A). Besides, oncogenic stress can cause DNA damage mediated by mitochondrial ROS^19,43,44^. Moreover, mitophagy is an important mechanism to prevent the surge/increases of ROS clearing-damaged mitochondria^22,45–47^. Thus, we proposed that in E6E7 keratinocytes under HRas^G12V^ activity, autophagy could be acting in the clearance of damaged mitochondria softening the deleterious effects of ROS^47,48^.

The context dependence of autophagy in cancer cells has been largely discussed^22,49–52^. Some authors attribute to autophagy a potential for cell death driving^38,53^; however, in most cases where autophagy is associated with cell death, the mechanism is activated as a prosurvival attempt^54^. Here, we are proposing that in E6E7 keratinocytes, autophagy was recruited as a prosurvival mechanism to mitigate the oncogenic stress caused by HRas^G12V^ activity; however, only in moderate oncogenic activity, the mechanism can support the transition from immortalized to the malignant transformed phenotype.

This is a convincing proposition when we consider that E6E7 keratinocytes were unviable under any level of HRas^G12V^ activity when autophagy was blocked (Fig. 6). Despite the essential role of autophagy in keratinocytes transitioning from immortalized to malignant state, its intense and continuous recruitment overloaded the system causing an uncontrolled flux (Figs. 5B and 6D), demonstrated by the accumulation of p62 (Figs. 5A and 6A/D). The accumulation of p62 by itself is highly toxic, causing even more generation of ROS, which might contribute to the phenotype observed^55,56^. Therefore, we concluded that autophagy is initiated as a protective mechanism increasing cell survival; however, sustaining intense autophagic induction caused by acute ROS surges generates an autophagic flux beyond the cell processing capacity. As a result, autophagy became an additional source of stress.

Finally, cells exposed to HRas^G12V^ activity can be aided by metabolic reprogramming increasing the glycolytic activity through the MAPK and PI3K axes. This metabolic reprogramming helps in the generation of biomass such as amino acids and nucleotides^57^. Our results showed that E6E7 keratinocytes under HRas^G12V^ activity highly signaled MAPK and PI3K, and might be responsible for a metabolic shift (Figs. 1A, 2E, 4A, and 6D). In addition, the increased concentration of lactate in the cell culture medium suggested that E6E7 keratinocytes under high HRas^G12V^ activity were pushed to reprogramming its metabolism to aerobic glycolysis. Likewise, the elevated mitogenic signal could increase the number of DNA replication origins fired, further stressing the limited levels of deoxynucleotide pools causing genomic instability and consequent loss of cell viability^58^. As shown in Fig. 7D, treatment with deoxynucleosides partially rescued the viability of induced keratinocytes.

In conclusion, our results showed that high levels of HRas^G12V^ activity are not tolerated by E6E7 keratinocytes under any circumstances for two reasons: first, deleterious levels of mitogenic, replication, and ROS stresses responsible for high genotoxicity; second, recruitment and activation of autophagy beyond the processing capacity of the cell system, causing accumulation of toxic content not properly processed (Fig. 8). These results per se are proposing an alternative molecular approach to defeat malignantly transformed cells, that consists in the imposition of even higher levels of mitogenic signals while autophagy is blocked, which could be a promising therapeutic strategy when limiting cell growth using mitogen-activated protein kinase inhibitors proves to be ineffective or refractory.

## Methods

### Transduction, parental cells, and sublineage cultures

All parental cells and sublineages listed below were grown in *KSFM* medium (Thermo Fisher^*®*^) supplemented with recombinant epidermal growth factor 5 ng/ml, bovine pituitary extract 50 mg/ml (except when otherwise indicated) and incubated at 37 °C in 5% CO_2_ atmosphere.

### Immortalization of primary human keratinocytes with E6E7–HPV16 proteins

Primary foreskin human keratinocytes (PHK) (Lonza Walkersville, Inc., Walkersville, MD) were transduced with pLXSN-encoding E6E7–HPV16 proteins. After 24 h of acute transduction, transduced keratinocytes were selected using 300 µg/mL of G418. The retrovirus pLXSN was kindly provided by Dr. Denise Galloway (Fred Hutchinson Cancer Research Center, Seattle, WA), which was previously described^59^.

### Constitutive (SLcs) and inducible (SLis) keratinocyte sublineages

PHK immortalized by E6E7/HPV16 proteins was transduced with retrovirus constructs for 48 h. pBabepuro-HRas^G12V^ was utilized for constitutive expression (SLcs) and pBabepuro-ER:HRas^G12V^, for inducible expression sublineages (SLis) (Supplementary Fig. S1). After this period, transduced cultures were selected using 2.5 µg/mL of puromycin (Invitrogen^©^). Three independent clones were generated for each sublineage. Additionally, two independent empty controls were generated pBabe-ø and pBabe-ER:ø for constitutive and inducible sublineages, respectively. Concentrations from 5 nM to 100 nM of 4OHT proved to be innocuous in the parental E6E7 keratinocytes and ER:ø-keratinocytes (Supplementary Fig. S1 J/L) emphasizing that the results obtained are exclusively related to HRas^G12V^ activity and not the inductor 4OHT. All treatments, concentrations, and vehicles of dilution (used in the controls) are indicated in Supplementary Table 1.

### Cas9-mediated ATG7 knockout

To knock out the ATG7 gene in ER:HRas^G12V^ keratinocytes, we designed and tested two different gRNAs (ΔATG7I and ΔATG7II) using the CRISPR design tool (http://crispr.mit.edu/). A control gRNA (Scrb keratinocytes) that normally expresses ATG7 was also designed. The sequences are presented in Supplementary Table 1. Oligos were cloned into the LentiCRISPR v2 plasmid (a gift from Feng Zhang, Addgene plasmid # 52961) as described in Sanjana et al.^60^. For lentivirus production, LentiCRISPR v2 constructs, psPAX2 (a gift from Didier Trono, Addgene plasmid # 12260), and pCMV-VSV-G (a gift from Bob Weinberg, Addgene plasmid # 8454) were transfected into HEK293T cells using lipofectamine 3000 reagents according to the manufacturer’s protocol. Forty-eight hours after transfection, viral supernatant was collected, filtered, and applied to ER:HRas^G12V^ keratinocytes after the addition of 8 µg/ml polybrene. The knockout was tested after 7 days since both gRNAs showed a high efficiency; all the experiments were performed using the sublineage ER:HRas^G12V^ keratinocyte–ΔATG7I, hereafter ΔATG7 keratinocytes.

### Growth curves and cell saturation density assay

For growth curve assays, 7 × 10^3^ cells/cm^2^ were plated (except when differently stated) in Costar^®^ six-well plates. Forty-eight hours later, a six-well plate was collected to determine P0. This procedure was done to eliminate the lag phase of these cells and observe the effects of treatment and/or induction in the exponential phase. For maximum cell density saturation assays, 20 × 10^3^ cells/cm^2^ were plated. After 10 days (P10), the cultures reached their saturation density. The induction using 4-hydroxytamoxifen (4OHT) was initiated in P0, for the growth curve, and P10, for cell saturation density assays. In the days indicated cells were harvested using trypsin and resuspended in PBS + 5% fetal bovine serum (FBS) to inactivate the trypsin, washed, and fixed in a solution of phosphate buffer (PBS) with 3.7% of formaldehyde. All treatments and fresh medium were changed every other day. The cells were counted in Beckman Counter Z2^®^. The graphs present the results of at least two biological replicates in which three technical replicates for each treatment were collected.

### Western blots

The cells were plated in 60-mm^2^ Corning^®^ dishes and treated according to each experiment. The total protein was extracted and isolated in RIPA buffer (Sigma-Aldrich^®^) plus Halt^TM^ Protease Inhibitor Cocktail (Thermo Fisher^*®*^). The samples were quantified using Precision Red^TM^ (Cytoskeleton^*®*^) and submitted to western blot. Western blots were performed by standard chemiluminescence method using SuperSignal^®^ and Pierce ECL^®^ (Thermo Fisher^*®*^). The images were obtained by the UVITEC^®^ equipment and analyzed by the Alliance^®^ software. All the primary and secondary antibodies are listed in Supplementary Table 2.

### Pull down of Ras-GTP

Raf1 RBD agarose beads were incubated with 200 µg of the extract of total protein for 2 h at 4 °C in low rotation. Afterward, the beads attached to Ras-GTP were washed 8 times with PBS plus protease inhibitors. The beads were resuspended in NuPAGE^TM^ sample buffer (Thermo Fisher^*®*^) and applied to the acrylamide gel (12%). The western blot was performed using the anti-HRas antibody.

### Real-time PCR

Total RNA was extracted from cell culture by RNeasy kit (Qiagen^*®*^), quantified, converted to cDNA using MultiScribe RT kit (Thermo Fisher^®^), and submitted to real-time (RT)PCR. RT-PCR was carried out using the SYBR^®^ GREEN PCR Master Mix (Applied Biosystems™) combined to the primers listed in Supplementary Table 3 and the StepOne Plus PCR system. The results were calculated using the ΔΔCt method and then normalized by RPL19.

### Flow cytometry

For the assays performed in the flow cytometer, the cells were plated in Costar^®^ and proceeded as indicated by each experiment. All samples were run in the equipment Attune NxT Blue-Red (Thermo Fisher^®^) collecting 30000 events in at least two biological duplicates. The raw data were treated and analyzed by FlowJo^®^ V10.2.

### Cell cycle analysis

After the indicated period and treatment, cells were harvested and fixed in ice-cold 75% ethanol overnight at 4 °C. Fixed cells were washed twice in PBS, labeled with 100 µg/mL of propidium iodide (Thermo Fisher^®^), treated with 50 µg/mL of RNAse A (Invitrogen^®^) for 30 min, and then immediately submitted to flow cytometry. The quantification of single-labeled cell cycle phases was obtained by Watson pragmatic method^61^ using FlowJo^®^ V10.2. Cells were submitted to one hour of EdU pulse using the Click-iT^®^ Alexa Fluor 488 (Thermo Fisher^®^) according to the manufacturer’s recommendations for double-labeled cell cycle analysis.

### Cell viability assay

The cell viability was obtained from two methods as indicated in the experiments: sub-G1 population extracted from the cell cycle analysis and by double-labeling using Alexa Fluor® 488 annexin-V/PI Dead Cell Apoptosis Kit (Thermo Fisher^®^). The double-labeling was performed following the manufacturer’s recommendations.

### Assessment of ROS levels

The cells were plated in Costar^®^ six-well plates. After 48 h, the medium was replaced adding the treatments according to each experiment. The cells were incubated with 50 µM of 2′−7′-dichlorodihydrofluorescene diacetate (DCFDA Thermo Fisher^®^) for 1 h to measure general oxidative species, and 10 µM MitoSOX™ (Invitrogen^®^) for 30 min to assess the levels of mitochondria superoxide. After the incubation period, the cells were harvested and analyzed by flow cytometry.

### Measurement of lactate levels

The medium of 4OHT-induced and noninduced cells was collected every other day during the growth curve assessment. The media was centrifuged for 30 min at 14000 rpm in Centricon microtube cutoff 10 KDa (Vivaspin 500 GE Healthcare^®^). In total, 300 µL of the sample were collected in duplicates and kept at −80 °C until day 10, the last day of the experiment. The lactate concentration of all samples was measured at once following the manufacturer’s recommendations of the Lactate Assay Kit (Sigma-Aldrich^®^). The final concentration was normalized by the number of cells per cm^2^ of the well from where the medium was obtained.

### Immunofluorescence (IF)

For all experiments listed below, the images were captured using an Olympus BX51 fluorescence microscope coupled to a digital camera (XM10, Olympus), and were analyzed using Olympus- Cell F software (version 5.1.2640).

### Analysis of DNA breaks using TUNEL assay

To evaluate the presence of DNA break and compare the keratinocyte sublineages exposed to different concentrations of 4OHT with their respective controls, we used terminal deoxynucleotidyl transferase-mediated dUTP-Fluorescein nick-end-labeling (TUNEL) technique using the Apoptosis Detection System, Fluorescein kit (Promega). In total, 4 × 10^3^ cells/cm^2^ were plated onto the coverslips. After 48 h of treatment, the cells were washed with PBS and fixed using 1% paraformaldehyde for 10 min. The coverslips were washed with PBS and permeabilized by the addition of 0.1% Triton X-100 for 10 min at 26 °C. In all, 0.1 M glycine was added for 5 min to neutralize the remaining aldehyde groups. TUNEL assay was performed according to the manufacturer’s protocol. VECTASHIELD^®^ Mounting Medium with DAPI (Vector Labs) was added to be used as an antifade mounting solution and to stain the nuclei.

### Analysis of replication stress by BrdU native detection assay

For detection of long fragments of single-stranded DNA (ssDNA), a feature of replication stress, we grow keratinocytes in coverslips [induced with 50 nM 4OHT, noninduced (control), treated and nontreated with 5 nM NAC] in the presence of 50 mM 5-bromo-2’-deoxyuridine (BrdU) for 24 h to allow its incorporation into DNA. After that, we washed the coverslip-containing cells using PBS and fixed them using 4% of paraformaldehyde diluted in PBS for 10 min at room temperature. Next, cells were washed with PBS and permeabilized with 0.2% Triton-X 100 for 10 min at room temperature. To ensure that all cells incorporated BrdU, replicates of each condition were subjected to DNA denaturation using 2 M HCl. Then, all samples were washed, and BrdU was detected (when accessible) using α-BrdU-rat (Abcam) for 3 h at room temperature, followed by a 3-h incubation with the secondary antibody Alexa Fluor 555-conjugated goat anti-rat (Thermo Scientific). After that, the coverslip-containing cells were washed using 1× PBS and deposited on slides. VECTASHIELD^®^ Mounting Medium with DAPI (Vector Labs) was used to be an antifade mounting solution and to stain nuclear DNA.

### Statistical analyses

Two-way or one-way ANOVA followed by Bonferroni multiple-comparison test was employed when appropriate as indicated in figure legends. The data are presented as mean ± SD. The significance is pointed as the following: *P ≤ 0.05, **P ≤ 0.01, and ***P ≤ 0.001. All statistical analyses and graphs were prepared using *GraphPad Prism 7* software. In flow cytometry assays, the difference of the population distribution was calculated by the Kolmogorov–Smirnov statistic (K–S) in FlowJo^®^ V10.2.

## Supplementary information

Supplementary figure 1

Supplementary figure 2

Supplementary figure 3

Supplementary figure 4

Supplementary figure 5

Supplementary figure 6

Supplementary figure 7

Supplementary figure legend

Supplementary table 1

Supplementary table 2

Supplementary table 3
