# Correction: Autophagy buffers Ras-induced genotoxic stress enabling malignant transformation in keratinocytes primed by human papillomavirus

**DOI:** 10.1038/s41419-021-03564-4

**Published:** 2021-03-17

**Authors:** Eduardo Cararo-Lopes, Matheus H. Dias, Marcelo S. da Silva, Julianna D. Zeidler, Alexandre T. Vessoni, Marcelo S. Reis, Enrique Boccardo, Hugo A. Armelin

**Affiliations:** 1 Center of Toxins, Immune-response and Cell Signaling, Instituto Butantan, SãoPaulo, SP 05503-900 Brazil; 2 Department of Biochemistry, Instituto de Química, Universidade de São Paulo, São Paulo, SP 05508-000 Brazil; 3 Department of Chemical and Biological Sciences, Instituto de Biociência, Universidade do Estado de São Paulo, Botucatu, SP 18618-689 Brazil; 4 Department of Medicine, Washington University in St. Louis, St. Louis, MO 63110 USA; 5 Department of Microbiology, Instituto de Biociências, Universidade de São Paulo, São Paulo, SP 05508-900 Brazil; 6 Kogod Aging Center, Department of Anesthesiology and Perioperative Medicine, Mayo Clinic College of Medicine, Rochester, MN 55905 USA; 7 Present Address: Rutgers Cancer Institute of New Jersey, New Brunswick, NJ 08901 USA

**Keywords:** Cancer models, Macroautophagy, Stress signalling, Mechanisms of disease

Correction to: *Cell Death & Disease*

10.1038/s41419-021-03476-3 published online 18 February 2021

The original version of this article unfortunately contained an error in the affiliations. The affiliations were not correctly assigned to the authors. The original article has been corrected.

Eduardo Cararo-Lopes^1,2,7^*, Matheus H. Dias^1^, Marcelo S. da Silva^1,3^, Julianna D. Zeidler^1,6^, Alexandre T. Vessoni^4^, Marcelo S. Reis^1^, Enrique Boccardo^5^ and Hugo A. Armelin^1,2^*

Correspondence: Eduardo Cararo-Lopes (edu.llopes@gmail.com) or Hugo A. Armelin (haarmelin@iq.usp.br)

^1^Center of Toxins, Immune-response and Cell Signaling, Instituto Butantan, São Paulo, SP 05503-900, Brazil

^2^Department of Biochemistry, Instituto de Química, Universidade de São Paulo, São Paulo, SP 05508-000, Brazil

^1^Center of Toxins, Immune-response and Cell Signaling, Instituto Butantan, São Paulo, SP 05503-900, Brazil. ^2^Department of Biochemistry, Instituto de Química, Universidade de São Paulo, São Paulo, SP 05508-000, Brazil. ^3^Department of Chemical and Biological Sciences, Instituto de Biociência, Universidade do Estado de São Paulo, Botucatu, SP 18618-689, Brazil. ^4^Department of Medicine, Washington University in St. Louis, St. Louis, MO 63110, USA. ^5^Department of Microbiology, Instituto de Biociências, Universidade de São Paulo, São Paulo, SP 05508-900, Brazil. ^6^Kogod Aging Center, Department of Anesthesiology and Perioperative Medicine, Mayo Clinic College of Medicine, Rochester, MN 55905, USA. ^7^Present address: Rutgers Cancer Institute of New Jersey, New Brunswick, NJ 08901, USA.

