# Supplementary figures and images for: Autophagy buffers Ras-induced genotoxic stress enabling malignant transformation in keratinocytes primed by human papillomavirus
Source: Cell Death Dis. 2021 Feb 18;12(2):194. doi: 10.1038/s41419-021-03476-3 (PMC7892846; doi:10.1038/s41419-021-03476-3)

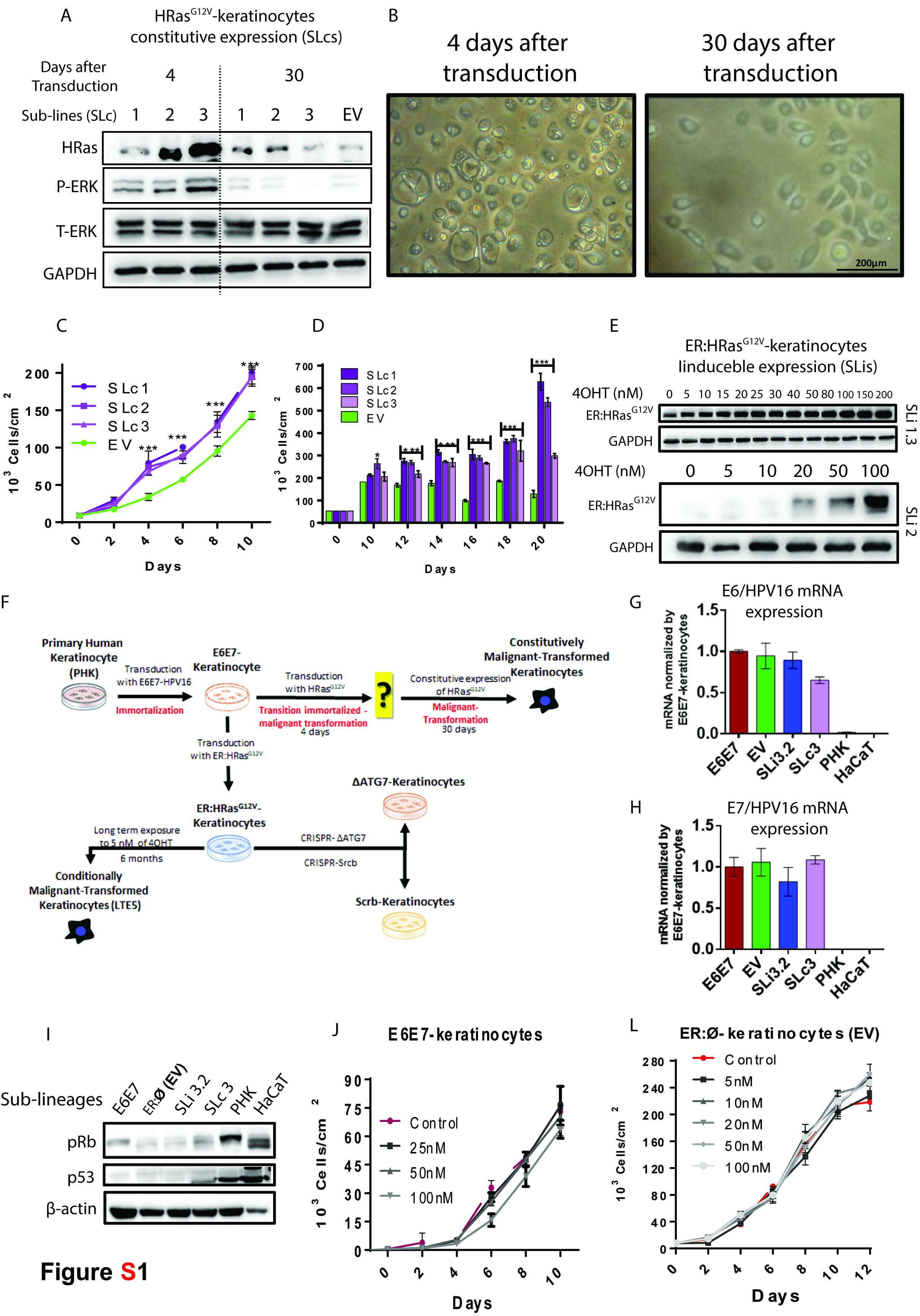

Supplement: Supplementary file 1 — Supplementary figure 1 [file 41419_2021_3476_MOESM1_ESM.tif]

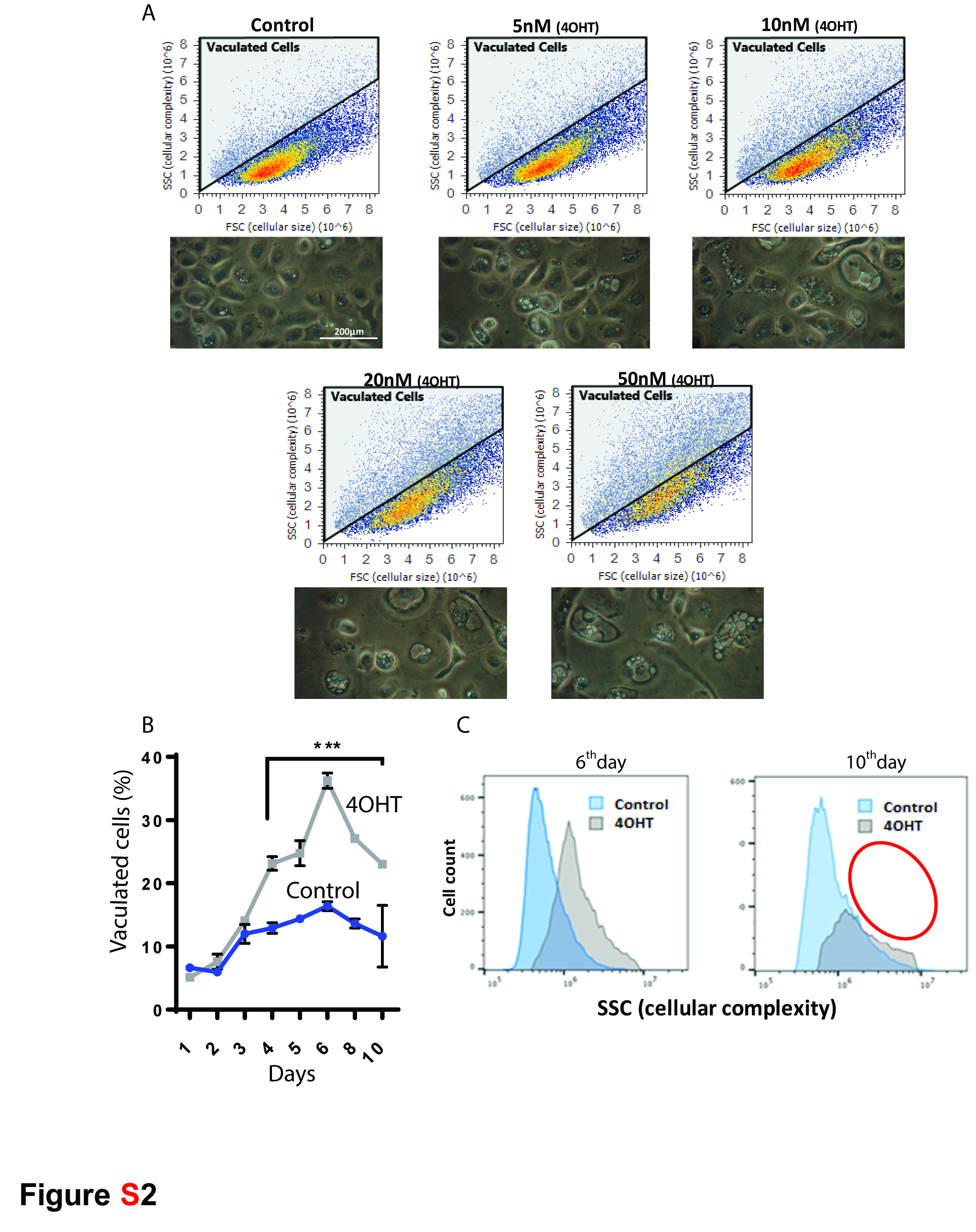

Supplement: Supplementary file 2 — Supplementary figure 2 [file 41419_2021_3476_MOESM2_ESM.tif]

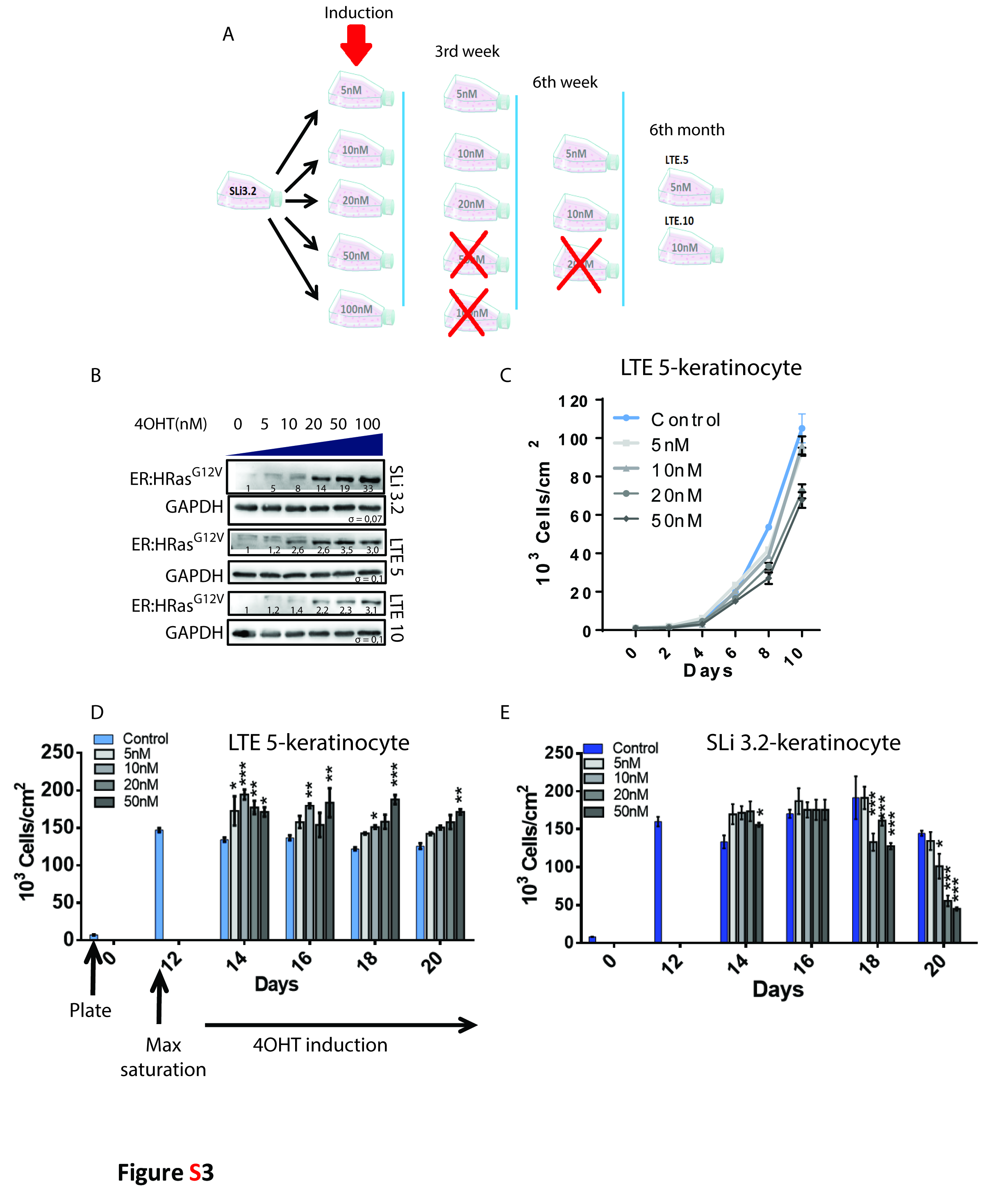

Supplement: Supplementary file 3 — Supplementary figure 3 [file 41419_2021_3476_MOESM3_ESM.tif]

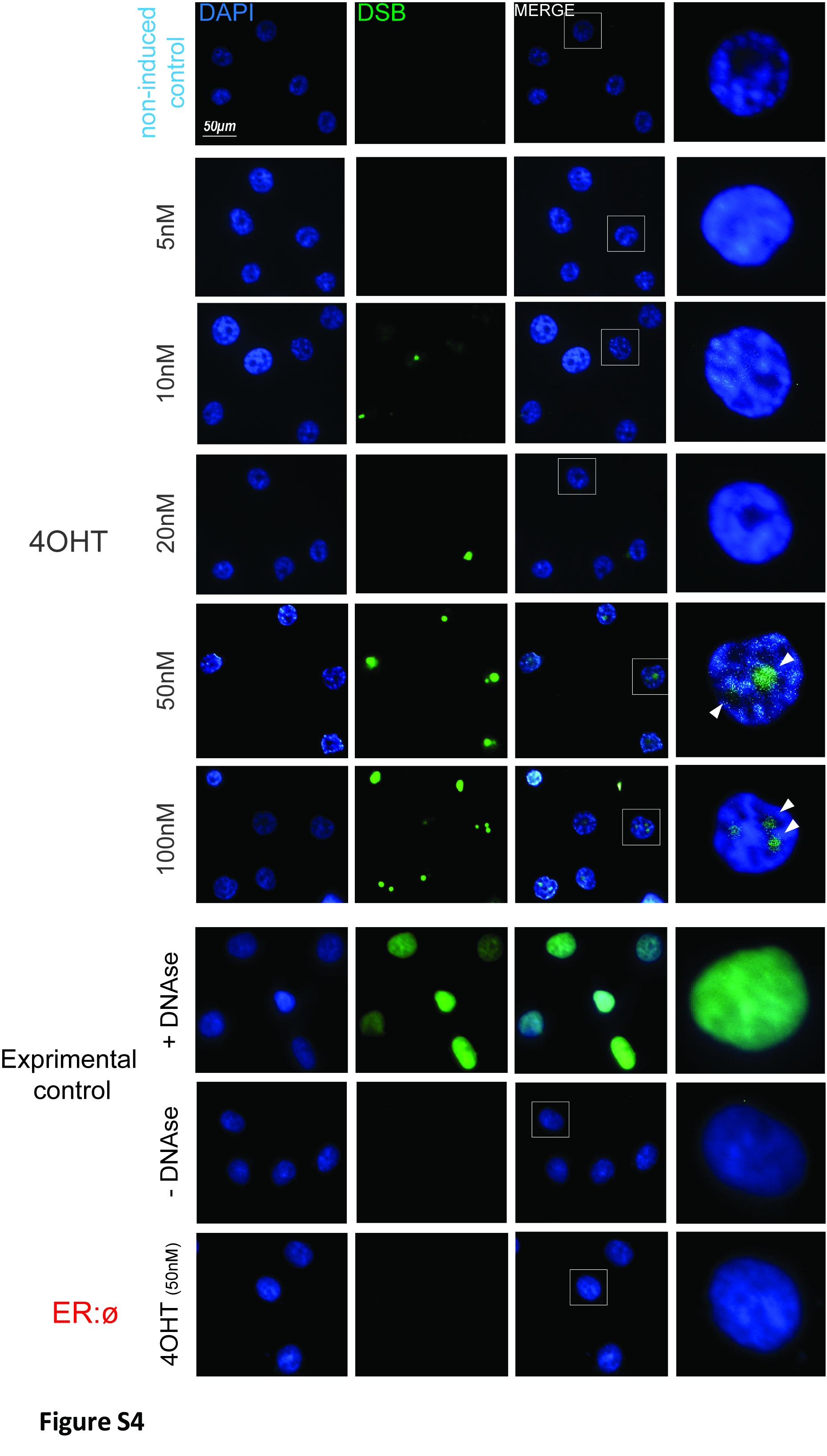

Supplement: Supplementary file 4 — Supplementary figure 4 [file 41419_2021_3476_MOESM4_ESM.tif]

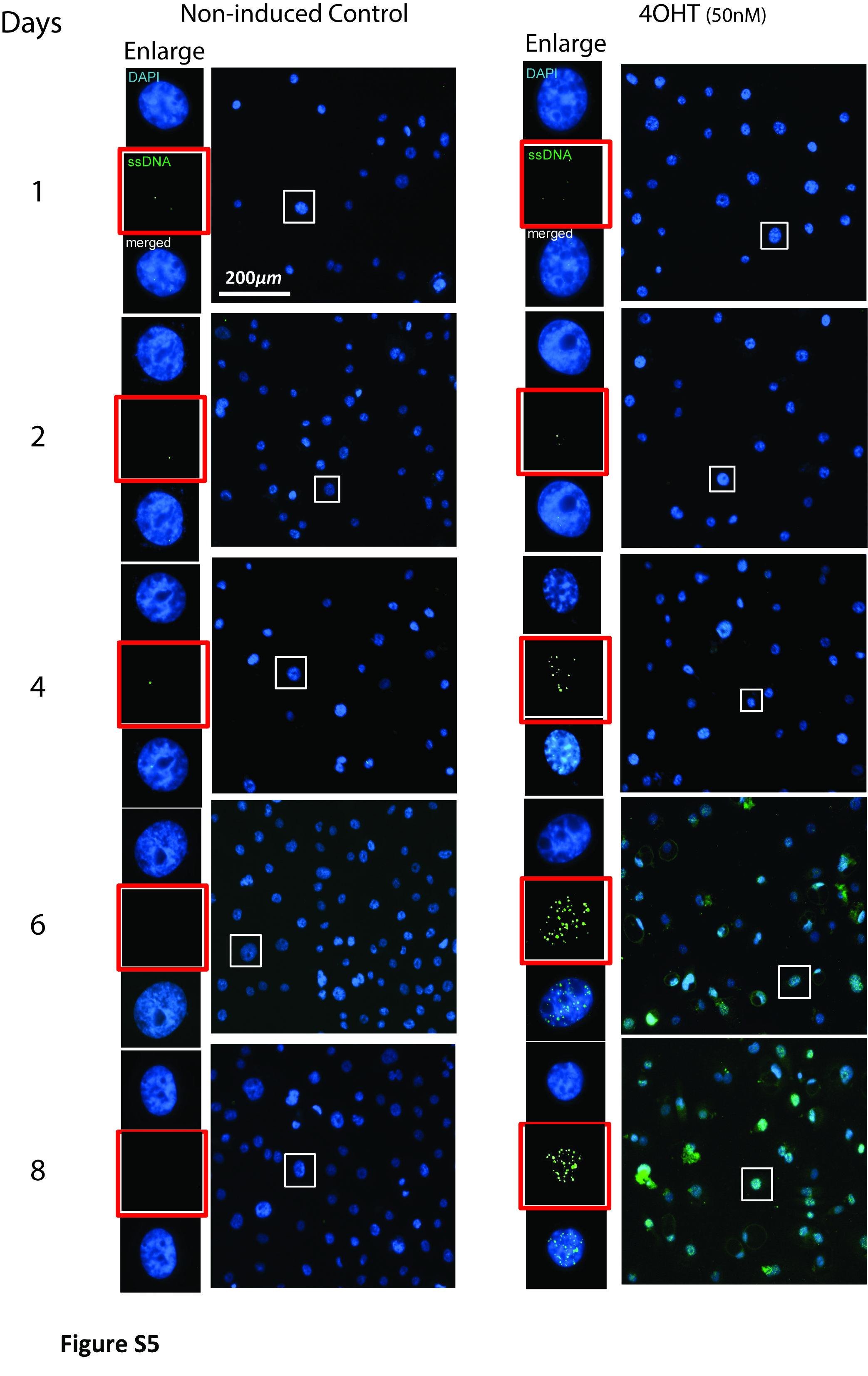

Supplement: Supplementary file 5 — Supplementary figure 5 [file 41419_2021_3476_MOESM5_ESM.tif]

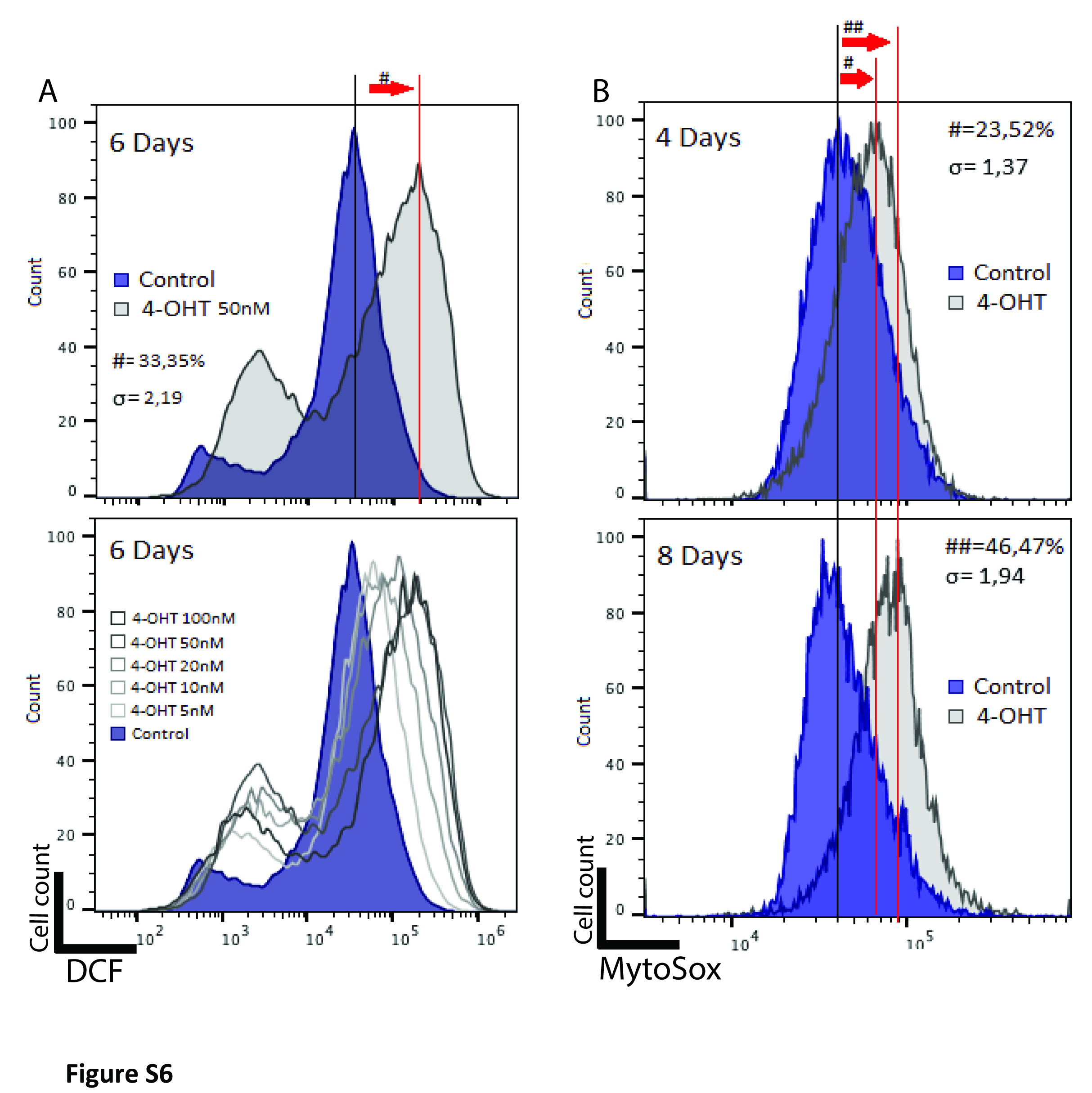

Supplement: Supplementary file 6 — Supplementary figure 6 [file 41419_2021_3476_MOESM6_ESM.tif]

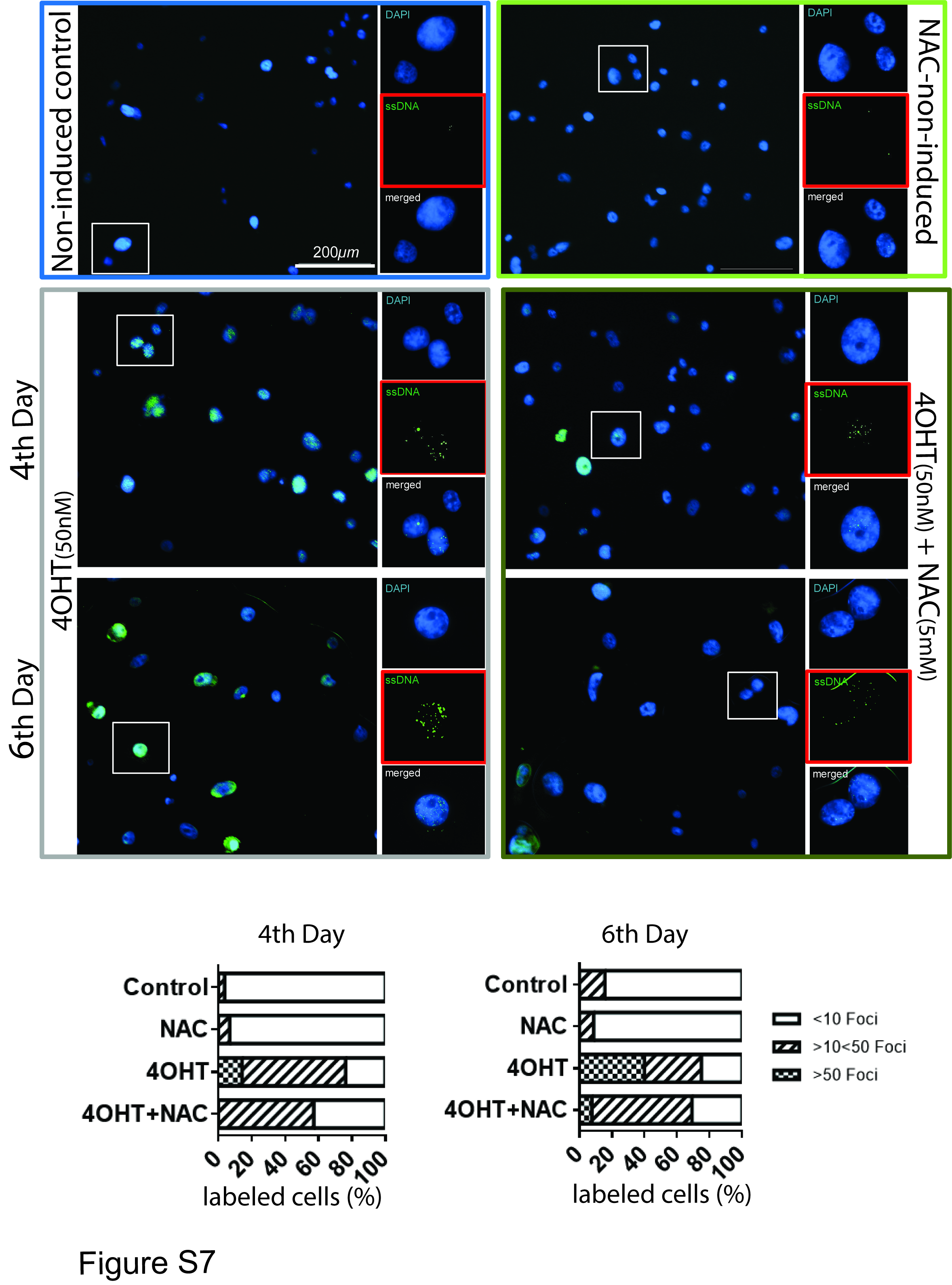

Supplement: Supplementary file 7 — Supplementary figure 7 [file 41419_2021_3476_MOESM7_ESM.tif]
