# Supplementary material for: Autophagy buffers Ras-induced genotoxic stress enabling malignant transformation in keratinocytes primed by human papillomavirus
Source: Cell Death Dis. 2021 Feb 18;12(2):194. doi: 10.1038/s41419-021-03476-3 (PMC7892846; doi:10.1038/s41419-021-03476-3)
Supplement: Supplementary file 8 — Supplementary figure legend [file 41419_2021_3476_MOESM8_ESM.docx]

**Supplementary Figure Legends**

**Supplementary Fig.S1: Characterization of keratinocyte-models generated.** (*A*) Immunoblots comparing three different constitutive sub-lineages (SLc) of HRas^G12V^-keratinocytes (SLc1, SLc2, and SLc3), generated independently, four, and thirty days after the transduction with the oncogene. The levels of HRas^G12V^, and its downstream pathway P-ERK, showed a negative selection of keratinocytes that were expressing high levels of HRas^G12V^-activity. (*B*) Light microscopy images of HRas^G12V^-constitutive sub-lineage SLc3 showing keratinocyte morphology four and thirty days after the constitutive HRas^G12V^ transduction. All constitutive cultures generated passed through a period of instability until the cells with lower levels of HRas^G12V^-activity were selected, given origin to keratinocytes with the same parental morphology but faster proliferative growth (*C*) and higher saturation density (*D*) in comparison with empty vector control, which are characteristics of keratinocytes in transition to malignant transformed phenotype. The graphs are representative of three independent experiments carried out in triplicate. (*E*) Immunoblotting of dose-response curve of 4OHT in two different inducible ER:HRas^G12V^–keratinocytes sub-lineages (SLi1.3 and SLi2). (*F*) Schematic view of the sub-lineages generated and their derivatives. The signal of “?” is in focus to represent the main goal of this article that is to reveal the mechanisms and events behind of the process of malignant transformation in HPV-primed keratinocytes. (*G/H*) qPCR showing the expression of HPV16/E6 and HPV16/E7 mRNAs levels in parental cells (E6E7), ER:Ø-keratinocytes empty vector (EV), ER:HRas^G12V^-keratinocyte inducible sub-lineage 3.2 (SLi3.2), HRas^G12V^-constitutive sub-lineage 3 (SLc3) and their negative controls PHK and HaCaT. (*I*) Immunoblots for p53 and pRb. The western blots show the decreased levels of p53 and pRb in the sub-lineages (E6E7, EV, SLi3.2, and SLc3) compared with the primary (PHK) and negative control (HaCaT) keratinocytes. This procedure emphasizes that the activity of E6 and E7 is responsible for degrading p53 and pRb, respectively. (*J/L*) The cytotoxicity of the inductor 4OHT was tested in parental (E6E7) and ER:Ø-keratinocytes empty vector (EV) showing to be innocuous in concentrations from 5 nM to 100 nM of 4OHT. The graphs are representative of three independent experiments carried out in triplicate. Data presented as a mean (SD). *Two-way ANOVA* (**) *p* ≤ 0.01 and (***) *p* ≤ 0.001, Bonferroni *posthoc*.

**Supplementary Fig.S2: Increased levels of HRas^G12V^-activity cause cytoplasmic vacuolation.**  (A) Cell complexity analysis by Flow Cytometry (upper image) and light microscopy (bottom image). In flow cytometry, cell complexity is detected by an increased SSC (side scatter) population, which corresponds to cell vacuolization/complexity observed by light microscopy. (*B*) Fixing the induction in 50 nM of 4OHT per ten days, we could follow the increase in cell complexity until the sixth day of induction. After this period, the most vacuolated keratinocytes population declines abruptly. The graph is representative of three independent experiments carried out in triplicate. (*C*) Histogram graph comparing 4OHT induced and non-induced (control) population in the sixth and tenth days, suggests that the absent population in this period was the more complex (vacuolated) population. Data presented as a mean (SD). *Two-way ANOVA* (**) *p* ≤ 0.01 and (***) *p* ≤ 0.001, Bonferroni *posthoc*.

**Supplementary Fig.S3: Only moderate HRas^G12V^-activity is capable of generating malignant cell characteristics in immortalized E6E7-keratinocytes.** (A-E) Generation of long-term exposure (LTE) sub-lineages 5 and 10 showed that E6E7-keratinocytes can be malignant transformed only trough low HRas^G12V^ -activity. (*A*) We cultured the ER:HRas^G12V^-keratinocyte SLi 3.2 in six parallel groups: non-induced (control) and induced with 5, 10, 20, 50, and 100 nM of 4OHT. The complete medium was changed every other day, and induced groups had the 4OHT replaced in the same concentration until the end of the experiment. After three weeks, all the cells induced with 50 or 100 nM of 4OHT had died. After six weeks, the same happened with cells induced with 20 nM of 4OHT. After six months of the experiment, only non-induced (SLi 3.2), 5 nM (LTE 5), and 10 nM (LTE 10) induced ER:HRas^G12V^-keratinocyte had survived. (*B*) Immunoblots comparing three different sub-lineages suggest that LTE5- and LTE10-keratinocytes lost their potential to induce higher levels of HRas^G12V^ expression. The immunoblotting quantification are presented below each band. All 4OHT induced samples are normalized by the non-induced control (first band in the immunoblotting). The pattern deviation of housekeepers is expressed by the letter σ. (*C*) As a result of this lower levels of 4OHT induction, LTE-keratinocytes can grow in 4OHT concentrations from 5 to 50 nM, differently from what is observed in the parental ER:HRas^G12V^-keratinocyte (Figures 1 and 2). (*D*) 12 days after seeding keratinocytes in a concentration of 10^4^ cells/cm^2^, the maximum saturation density is reached. From days 12 to 20, we induced LTE-keratinocytes with different 4OHT concentrations in parallel with the non-induced control. All 4OHT concentrations used were capable to increase the maximum saturation density of LTE-keratinocytes. (*E*) An identical experiment using the parental ER:HRas^G12V^-keratinocyte was conducted and showed the opposite result of LTE-keratinocytes. The graphs are representative of two independent experiments carried out in triplicate. *Two-way ANOVA* (**) *p* ≤ 0.01 and (***) *p* ≤ 0.001, Bonferroni *posthoc*.

**Supplementary Fig.S4: DNA strand breaks (DSB) increase in a dose-dependent manner of HRas^G12V^-activity.**  TUNEL assay for ER:HRas^G12V^-keratinocytes induced for six days with different 4OHT concentrations and their respective controls. In the first line (written in blue), the non-induced control of sub-lineage ER:HRas^G12V^-keratinocyte is shown. Written in grey DSBs *foci* induced in a 4OHT dose-dependent manner. In addition to non-induced ER:HRas^G12V^-keratinocyte three additional controls were used: Controls treated and non-treated with DNase (in order to show that the experiment was working for all conditions) and control using the sub-lineage ER:∅-keratinocytes treated with 50 nM of 4OHT for six days (to show that the inductor is not responsible for the DSB observed). The quantification of those entire images is presented in figure 3B.

**Supplementary Fig.S5: Kinects of ssDNA accumulation caused by high HRas^G12V^-activity.** Images of whole kinetics of native BrdU assay as presented in the graph shown in Fig. 3A. ER:HRas^G12V^-keratinocytes were induced with 50 nM of 4OHT for eight days. A matched non-induced (control) is also presented for each time point. Cells marked with a white square in the panoramic microscopy image are enlarged on the side, showing DAPI (blue) and ssDNA (green) *foci*.

**Supplementary Fig.S6: High HRas^G12V^-activity increases ROS levels.** (A) (Top) The first graph shows ROS levels in ER:HRas^G12V^-keratinocyte with or without 50 nM of 4OHT induction for six days. The panel on the bottom shows the fluorescence of DCF under different concentrations of 4OHT induction. (B) Increased levels of mitochondrial superoxide measured by MitoSox. The ER:HRas^G12V^-keratinocytes started to present increased levels of mitochondrial superoxide after four days of induction with 50 nM of 4OHT (top) and achieved its maximal levels after eight days (bottom). The dislocation of the fluorescent population, relative to its non-induced control, was measured by the *Kolmogorov-Smirnov test (K-S).* The distances were pointed in the percentage of displacement and indicated through the symbol # or ## as indicated in the figures. The σ symbol expresses the standard deviation between three independent experiments.

**Supplementary Fig.S7: NAC treatment counteracts the ssDNA exposure caused by high HRas^G12V^-activity.** The composition in the upper panel shows the controls [non-induced control (blue box) and non-induced control treated with 5 mM of NAC (light green box)] on the sixth day. In the lower panel, it is presented ER:HRas^G12V^-keratinocytes induced with 50 nM of 4OHT treated (dark green box) or not treated (grey box) with 5 mM of NAC in the 4^th^ and 6^th^ days. The red squares are presenting enlarged pictures (10x) of the nuclei to emphasize the decreased number of ssDNA *foci* in NAC treated keratinocytes. For each condition and days, at least 50 nuclei were counted and categorized in three different phenotypes: normal (≤ 10 *foci* per nucleus); early stage of replication stress (≥ 10 ≤ 50 *foci* per nucleus), and advanced stage of replication stress (≥ 50 *foci* per nucleus).
