# Supplementary material for: Autophagy buffers Ras-induced genotoxic stress enabling malignant transformation in keratinocytes primed by human papillomavirus
Source: Cell Death Dis. 2021 Feb 18;12(2):194. doi: 10.1038/s41419-021-03476-3 (PMC7892846; doi:10.1038/s41419-021-03476-3)
Supplement: Supplementary file 9 — Supplementary table 1 [file 41419_2021_3476_MOESM9_ESM.docx]

**Supplemental Table 1:** Reagents used to treat cell cultures.

| **Reagent** | **Manufacturer** | Code | Concentration used | Solvent |
| --- | --- | --- | --- | --- |
| 4-Hydroxytamoxifen | Sigma-Aldrich® | H7904 | 5, 10, 20, 50 and 100 nM | Ethanol |
| N-Acetyl-L-cysteine (NAC) | Sigma-Aldrich® | A7250 | 5 mM | H2O |
| Hydroxychloroquine | Sigma-Aldrich® | H0915 | 5 µM | H2O |
| Deoxyadenosine | Sigma-Aldrich® | D8668 | 2 mM | H2O |
| Deoxyguanosine | Sigma-Aldrich® | D7145 | 2 mM | 1 M of NH4OH |
| Deoxycytidine | Sigma-Aldrich® | D3897 | 2 mM | H2O |
| Thymine | Sigma-Aldrich® | T0376 | 2 mM | 1 N NaOH |
