# Supplementary material for: Autophagy buffers Ras-induced genotoxic stress enabling malignant transformation in keratinocytes primed by human papillomavirus
Source: Cell Death Dis. 2021 Feb 18;12(2):194. doi: 10.1038/s41419-021-03476-3 (PMC7892846; doi:10.1038/s41419-021-03476-3)
Supplement: Supplementary file 10 — Supplementary table 2 [file 41419_2021_3476_MOESM10_ESM.docx]

**Supplemental Table 2:** Antibodies used in this article.

| **Anti-body** | **Code** | **Manufacturer** | **Method** |
| --- | --- | --- | --- |
| Akt | #9272 | Cell Signaling® | WB |
| Anti-BrdU | ab6326 | Abcam® | IF |
| Anti-SQSTM1 / p62 | ab56416 | Abcam® | WB-IF |
| ATG7/APG7 | sc-8668 | Santa Cruz® | WB |
| GAPDH | sc-47724 | Santa Cruz® | WB |
| ɣH2A.X (S139) | ab2893 | Abcam® | WB |
| Goat anti-rabbit conjugated with peroxidase | 474-1506 | KPL® | WB |
| Goat anti-mouse conjugated with peroxidase | 041-806 | KPL® | WB |
| Goat anti-Mouse IgG conjugated to Alexa Fluor Plus 488 | A32723 | ThermoFisher® | IF |
| Goat anti-Rabbit IgG conjugated to Alexa Fluor 568 | A11011 | ThermoFisher® | IF |
| Histone H2A.X | ab11175 | Abcam® | WB |
| HRas | sc-520 | Santa Cruz® | WB-PD-IF |
| LC3 | #3868 | Cell Signaling® | WB-IF |
| p16 | sc-1661 | Santa Cruz® | WB |
| p19 | sc-1063 | Santa Cruz® | WB |
| p21 | sc-6246 | Santa Cruz® | WB |
| p27 | sc-528 | Santa Cruz® | WB |
| p38 MAP Kinase | #9212 | Cell Signaling® | WB |
| p44/42 MAPK (Erk1/2) | #4695 | Cell Signaling® | WB |
| Phospho-Akt (Ser473) | #9271 | Cell Signaling® | WB |
| Phospho-p38 MAP Kinase (Thr180/Tyr182) | #9211 | Cell Signaling® | WB |
| Phospho-p44/42 MAPK (Erk1/2)(Thr202/Tyr204) | #9101 | Cell Signaling® | WB |
| Phospho-p53 (ser15) | #9285 | Cell Signaling® | WB |
| Rabbit anti-goat conjugated with peroxidase | 141-306 | KPL® | WB |
| WB-Western Blot; IF- Immunofluorescence; PD- Pull Down | | | |
