# Supplementary material for: Autophagy buffers Ras-induced genotoxic stress enabling malignant transformation in keratinocytes primed by human papillomavirus
Source: Cell Death Dis. 2021 Feb 18;12(2):194. doi: 10.1038/s41419-021-03476-3 (PMC7892846; doi:10.1038/s41419-021-03476-3)
Supplement: Supplementary file 11 — Supplementary table 3 [file 41419_2021_3476_MOESM11_ESM.docx]

**Supplemental Table 3:** Primers and guides used.

| **Target genes** | **Sequence 5'-3'** |
| --- | --- |
| **E6/HPV16** | **F:**GAACAGCAATACAACAAACCGT |
|  | **R:**AGGACACAGTGGCTTTTGAC |
| **E7/HPV16** | **F:**TTTGCAACCAGAGACAACTGAT |
|  | **R:**TTCATCCTCCTCCTCTGAGCTG |
| **RPL19** | **F:**TGGGCTGATCATCCGCAAGCC |
|  | **R:**CCCATGTGCCTGCCCTTCCG |
| **Scrb** | **Guide-**GCACTACCAGAGCTAACTCA |
| **ΔATG7 I** | **Guide-**TTGAAAGACTCGAGTGTGT |
| **ΔATG7 II** | **Guide-**CTCTTGTAAATACCATCTGT |
| F: Forward and R: Reverse | |
